# Predictors of Response to Biologics for Severe Asthma: A Systematic Review and Meta‐Analysis

**DOI:** 10.1111/all.70031

**Published:** 2025-09-16

**Authors:** Anna Rattu, Piers Dixey, David Charles, Chris Brightling, Kian Fan Chung, Apostolos Bossios, Arnaud Bourdin, Ratko Djukanovic, Sven‐Erik Dahlén, Louise Fleming, Rekha Chaudhuri, Erik Melén, Antoine Deschildre, Charles Pilette, Gerard H. Koppelman, Andrew Exley, Freja Anckers, Sarah Miller, Hanna Nielsen, Clare Williams, Ekaterina Khaleva, Graham Roberts, the 3TR consortium Respiratory Work Package

**Affiliations:** ^1^ Clinical and Experimental Sciences, Faculty of Medicine University of Southampton Southampton UK; ^2^ NIHR Southampton Biomedical Research Centre University Hospital Southampton NHS Foundation Trust Southampton UK; ^3^ National Heart Lung Institute Imperial College London UK; ^4^ Royal Brompton Hospital London UK; ^5^ Academic Clinical Medicine Southampton General Hospital Southampton UK; ^6^ Institute for Lung Health, Leicester NIHR BRC University of Leicester Leicester UK; ^7^ National Heart and Lung Institute Imperial College London London UK; ^8^ Karolinska Severe Asthma Center, Department of Respiratory Medicine and Allergy Karolinska University Hospital Stockholm Sweden; ^9^ Division of Lung and Airway Research, Institute of Environmental Medicine Karolinska Institute Stockholm Sweden; ^10^ Lung Laboratory, Center for Molecular Medicine Karolinska University Hospital Stockholm Sweden; ^11^ PhyMedExp University of Montpellier Montpellier France; ^12^ NIHR Southampton Biomedical Research Centre University Hospital Southampton Southampton UK; ^13^ Sir Henry Wellcome Laboratories Southampton UK; ^14^ Department of Respiratory Medicine and Allergy Karolinska University Hospital Huddinge Sweden; ^15^ Department of Medicine, Huddinge, Karolinska Institutet, and Institute of Environmental Medicine Karolinska Institutet Stockholm Sweden; ^16^ Imperial College Healthcare Trust and National Heart and Lung Institute Imperial College London UK; ^17^ School of Infection and Immunity University of Glasgow Glasgow UK; ^18^ Department of Clinical Science and Education Södersjukhuset Karolinska Institutet Stockholm Sweden; ^19^ CHU Lille, Unité de Pneumologie et Allergologie Pédiatrique Hôpital Jeanne de Flandre Lille France; ^20^ Univ. Lille U1019 – UMR 8204 – CIIL – Center for Infection and Immunity of Lille Lille France; ^21^ Department of Pulmonology, Cliniques Universitaires Saint‐Luc and Pole of Lung, Nose and Skin Research (LUNS) Institute of Experimental and Clinical Research (IREC) Brussels Belgium; ^22^ Department of Pediatric Pulmonology and Pediatric Allergology University of Groningen, University Medical Center Groningen, Beatrix Children's Hospital Groningen the Netherlands; ^23^ Groningen Research Institute for Asthma and COPD (GRIAC) University of Groningen, University Medical Center Groningen Groningen the Netherlands; ^24^ Adept Biologica Consulting Limited London UK; ^25^ Patient and Public Involvement Representative Sweden; ^26^ Patient and Public Involvement Representative UK; ^27^ Falu Lasarett, Region Dalarna Falun Sweden; ^28^ European Lung Foundation Sheffield UK; ^29^ Royal Hampshire County Hospital Winchester UK; ^30^ Human Development and Health, Faculty of Medicine University of Southampton Southampton UK; ^31^ Paediatric Allergy and Respiratory Medicine University Hospital Southampton NHS Foundation Trust Southampton UK

**Keywords:** biologics, predictive biomarkers, response, severe asthma

## Abstract

Biologics are effective for severe asthma, but not all patients benefit equally. There is an urgent need to understand which biologic works best for which patient. We systematically searched for predictors of response to biologics (except omalizumab) for severe asthma in four bibliographic databases and two trial registries from 1990 to 2024. Two reviewers screened records, extracted data, and assessed risk of bias using a modified CASP checklist. Data were synthesized narratively, and certainty of evidence assessed using the modified GRADE framework. Comparable studies were meta‐analyzed using a random‐effects model. From 5853 records, 21 studies were identified investigating predictors of anti‐IL5/5Rα, 4Rα, and anti‐TSLP response. We found predominantly ‘moderate’ to ‘high’ quality evidence that raised blood eosinophil counts (≥ 300 cells/μL), FeNO levels (> 40 ppb), lack of or low OCS dose (< 10 mg/day), and better asthma control predict biologic response. Evidence for the predictive value of other characteristics was limited and mostly ‘low’ quality. Key reasons for downgrading the evidence were heterogeneous response definitions and imprecision. No data were identified for the pediatric population or biologics targeting the non‐T2 pathway. Outside of traditional inflammatory and clinical variables, there is an unmet need for universally applicable predictors of biologic response for severe asthma.

**Abbreviations**
ACQ
: Asthma Control Questionnaire
ACT
: asthma control test
ATS/ERS
: American Thoracic Society/European Respiratory Society
AUC
: area under the curve
BEC
: blood eosinophil counts
BMI
: body mass index
CASP
: clinical appraisal skills programme
CI
: confidence intervals
COMSA
: core outcome measures set for paediatric and adult severe asthma
EMA
: European Medicines Agency
FeNO
: fractional exhaled nitric oxide
FEV_1_

: forced expiratory volume in 1 s
GERD
: gastroesophageal reflux disease
GRADE
: grading of recommendations assessment development and evaluation
ICS
: inhaled corticosteroids
IgE
: immunoglobulin E
IL
: interleukin
MCID
: minimal clinical important difference
MID
: minimal important difference
mOCS
: maintenance oral corticosteroids
NP
: nasal polyposis
PICO
: patient intervention, comparator and outcomes
ppb: parts per billion
PRISMA
: Preferred Reporting Items for Systematic Reviews and Meta‐Analyses
QoL
: quality of life
RCT
: randomised controlled trial
RoB
: risk of bias
SD
: standard deviation

## 1 Background

Severe asthma affects approximately 5% of the global asthma population [1], yet it imposes a disproportionate burden in terms of exacerbations requiring hospitalizations, impaired Quality of Life (QoL) [2, 3], and socioeconomic costs [4, 5]. Although most patients achieve adequate control with high‐dose inhaled corticosteroids (ICS) and additional controller therapies, a subset remains uncontrolled despite maximal pharmacotherapy [6]. For these patients, targeted biologics as add‐on treatments can reduce exacerbation frequency and oral corticosteroid (OCS) use while improving lung function and symptom control [1].

Biologics herald an era of personalised medicine for severe asthma, but they are expensive [7] and have associated side effects [8]. Pertinently, not all patients benefit equally, with non‐response rates ranging from 15% to 17% [9, 10, 11] and partial response rates from 43% to 69% [11, 12]. This can be attributed to factors such as heterogeneity at the pathophysiological and population level, and differences in treatment strategy [13]. Therefore, in lieu of a ‘one‐size‐fits all’ approach, it is necessary to identify the right biologic for the right patient i.e., prediction of response. Inflammatory biomarkers, such as raised fractional exhaled nitric oxide (FeNO) and high blood eosinophil counts (BEC) have been established as predictors of biologic response using trial registry data, and subsequently informed regulatory approval criteria and treatment guidelines [14, 15, 16]. However, these guidelines do not always align with real life, partly due to the homogenous populations included in the underpinning randomised controlled trials (RCTs). Additionally, composite predictors of biologic response have been explored [17, 18], including a tool to predict omalizumab response based on asthma control [19], though this has not been widely implemented as it lacks physiological markers crucial for gauging treatment benefit.

The pursuit of biologic response predictors is complicated by the heterogeneity in definitions of response [20]. These range from measurement of improvement in individual outcomes (e.g., exacerbations) to composites including multiple outcomes, cutoffs, and disparate nomenclature (e.g., non‐response, response, super‐response) [21, 22]. Inconsistency in the timepoints of evaluation further hampers the quest for response predictors, with some studies investigating potential predictors using baseline data collected pre‐biologic, while others use data from an early assessment several months after initiating therapy.

A meta‐analysis established that baseline BEC and total serum IgE levels can serve as reliable predictors of omalizumab response in patients with allergic asthma [23]. However, a comparable synthesis for other biologics is lacking. Therefore, this systematic review aimed to identify predictors of response to biologics (except omalizumab) for severe asthma.

## 2 Methods

This systematic review is registered with the International Prospective Register of Systematic Reviews (PROSPERO registration: CRD42022272057). The Preferred Reporting Items for Systematic Reviews and Meta‐Analyses (PRISMA) checklist has been used to guide the reporting of this systematic review [24] (Table S7).

### 2.1 Search Strategy

A search strategy was developed on EMBASE (OVID) and subsequently adapted for the following databases: MEDLINE (OVID), CINAHL (EBSCOhost, Cumulative Index to Nursing and Allied Health Literature), and ISI Web of Science (Thomson Web of Knowledge) (see Data S1). Databases were searched from 1990 to 10 January 2024. ClinicalTrials.gov (CT.gov) and International Trials Registry Platform (ICTRP) were also searched. The reference lists of identified studies, systematic reviews, reviews, and guidelines were searched for additional articles. Experts within the 3TR Respiratory Work Package were also consulted to identify additional relevant articles. The search strategy included terms for omalizumab as it was developed prior to the identification of the systematic review on predictors of omalizumab response [23]. As detailed in the subsequent section, records focused on omalizumab were excluded during the citation screening process.

### 2.2 Eligibility Criteria

The inclusion criteria to identify eligible studies were as follows:

*Population*: adults (≥ 18 years) and/or children (6–17 years) with severe asthma according to the American Thoracic Society (ATS)/European Respiratory Society (ERS) guideline [6]. Databases were searched for all asthma severities to capture any records not accurately coded with ‘severe’ asthma.

*Intervention*: biologics which are licenced or under registration for severe asthma (excluding omalizumab). Studies reporting on multiple biologics were considered for inclusion if data could be extracted per biologic or obtained from the authors.

*Comparator*: any comparator group, including placebo, active comparator, or no intervention.

*Predictors of biologic response*: any predictor such as inflammatory markers, lung function parameters, clinical parameters, co‐morbidities, socio‐demographic characteristics etc.

*Outcomes*: response definition including the COMSA outcomes [25, 26] (exacerbations, FEV_1_, maintenance OCS use, asthma control, and QoL). All types of response definitions were eligible, such as partial response, super‐response, clinical response, and non‐response etc.

*Study designs*: controlled studies, real‐world studies, RCTs, *post hoc* or pooled analyses with data not reported in original articles (*n* ≥ 20) published as full‐text articles or research letters in English. Clinical trial records were searched to identify associated published studies.

The exclusion criteria were as follows: systematic reviews and meta‐analyses, narrative reviews, discussion papers, commentaries, non‐research letters and editorials, abstracts only (e.g., conference papers), case studies, unpublished materials, animal studies, non‐asthma studies e.g., viral bronchiolitis, viral associated wheeze. RCTs of licensed therapies using routes and dosages outside of the current EMA approach to prescription were excluded. Studies reporting on biologic switching were also excluded unless the washout period was ≥ 6 months; the review team arbitrarily selected this timeframe given the lack of official guidelines.

### 2.3 Study Selection and Data Extraction

References were pooled and de‐duplicated in Endnote version X9 (Thomson Reuters, Philadelphia, PA), and subsequently uploaded to Rayyan [27] (rayyan.qcri.org). Record titles, abstracts, and full texts were screened independently by two reviewers (AR, PD) according to the predefined eligibility criteria. Data about study design, sample size, participant characteristics, intervention and comparator (where appropriate), administration regime, predictor variable(s), response outcome(s) and associated effect estimates were extracted into a pilot‐tested form by three reviewers (AR, PD, DC). The final extraction was cross‐checked. Any disagreements were resolved through discussion or involvement of the third reviewer (GR)

### 2.4 Quality Appraisal Strategy

The methodological quality and certainty of evidence was evaluated in a two‐step process by two independent reviewers (AR, PD). Any discrepancies were resolved through discussion, or arbitration by the third reviewer (GR). First, the Risk of Bias (RoB) was assessed using the Clinical Appraisal Skills Programme (CASP) checklist for clinical prediction rules (CPR) [28], which was modified to appraise studies investigating predictors of treatment response. Each study was assessed and assigned a rating of ‘yes’, ‘no’, or ‘unclear’ for selected domains. The RoB assessment was conducted by two independent reviewers (AR, PD). Any discrepancies were resolved through discussion, or arbitration by the third reviewer (GR). Second, the certainty of evidence was assessed using the modified Grading of Recommendations, Assessment, Development and Evaluation (GRADE) framework [29, 30, 31]. The quality of evidence was graded as ‘high’, ‘moderate’, ‘low’, or ‘very low’. The criteria specific to this review are provided in Table S1.

### 2.5 Analysis and Synthesis of Results

Results were summarized using narrative synthesis and descriptive tables. Predictor variables with heterogeneous definitions across included studies (e.g., different thresholds for high BEC) were grouped for the narrative summary but were downgraded during the appraisal as per the criteria in Table S1. Pooled data from selected studies were included in a meta‐analysis (MA) using a random‐effects model, conducted in the STATA statistical package (STATA/IC 16.0 for Windows). Only studies similar in terms of predictor and response definitions, intervention, and comparator were included. Eligible response definitions included the COMSA outcomes [25, 26] (exacerbations, FEV_1_, maintenance OCS use, asthma control, and QoL). All response definition types were considered for example, partial response, super‐response, clinical response, and non‐response. Discrepancies were addressed by grouping similar definitions together, such as combining studies reporting clinical response based on exacerbations and asthma control. The MA identified the biologic response predictive power of the investigated variables. Where insufficient data were available for the MA, study authors were contacted to request additional data. It was not possible to assess publication bias due to the limited number of eligible studies.

## 3 Results

### 3.1 Study Characteristics

**FIGURE 1:**
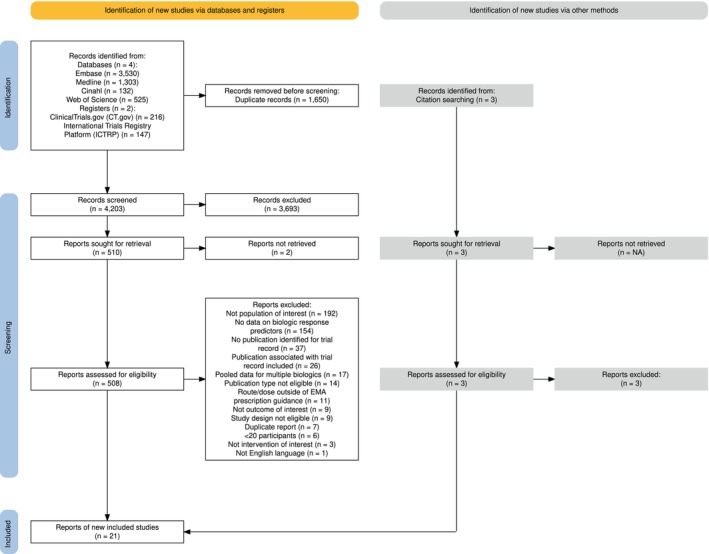
The database and registry search included omalizumab‐related terms, but records pertaining to omalizumab were excluded during screening. CT, clinical trials; EMA, European Medicines Agency; ICTRP, International Clinical Trials Registry Platform.

The study selection process is summarized in Figure 1. The search strategy yielded a total of 5853 records, with three additional records identified from the review of citations. Following the deletion of duplicate records, 4203 records were screened by title and abstracts and excluded if they did not meet the eligibility criteria. The full text of 508 records was assessed for eligibility; predominant reasons for exclusion were reporting of pooled data across mild‐to‐severe asthma, absence of data on predictors of biologic response, and lack of published data for clinical trial records. Ultimately, 13 studies were identified for mepolizumab [9, 32, 33, 34, 35, 36, 37, 38, 39, 40, 41, 42], 6 for benralizumab [33, 43, 44, 45, 46, 47], 3 for dupilumab [48, 49, 50], and 1 for tezepelumab [51]. The characteristics of included studies are presented in Table 1. Most studies were real‐world studies conducted in multinational settings. The sample sizes of the included studies ranged from 20 to 822 participants, with participant ages spanning from 28 to 82 years. None of the identified studies investigated response predictors for pediatric severe asthma, or biologics targeting the non‐T2 pathway.

**TABLE 1:** Characteristics of included studies.

Reference, year	Country	Study design	*N* total (treated—comparator)	Age (year), mean (SD) or median (range/IQR)	Participant characteristics	Characteristics of asthma	Administration regime
*Mepolizumab*							
Bergantini, 2020 [32]	Italy	Prospective, observational, real‐world study	20	56.3 (11.8)	M%: 35. Current/ex‐smokers %: 60.0	FEV_1_% (SD): 84.4 (26.6). BEC cells/mm^3^/%: 915 (617)/11.4 (6.5)	SC 100 mg/4 weeks
Bilò, 2023 [33]	Italy	Multicentre real‐world study	145	58.0 (11.0)	M%: 45. BMI kg/m^2^: 29.3 (30.5). Current/ex‐smokers %: 2.8/25.5	Exacerbation rate/y: 2.6 (2.8). Pre‐BD FEV_1_% pred.: 73.2 (19.9). Post‐BD FEV_1_% pred.: 77.7 (25.2). Eosinophils/mm^3^: 711.3 (1360.9). FeNO ppb: 42.0 (32.4). IgE values: 822.3 (2474.5). ACT score: 19.0 (4.7)	Not reported
Caminati, 2023 [34]^a^	Italy	Retrospective, observational study	68	Responder: 58 (53–67). Non‐responder: 52.5 (45–57)	Data presented by responders | non‐responders: M %: 42 | 30. BMI kg/m^2^, median (range): 24.1 (22.1–26.7) | 22.8 (20.8–27.0)	Median (Q1, Q3) presented by responders | non‐responders: FEV_1_% pred.: 64 (56, 81) | 63.5 (48, 77). BEC, μL^−1^: 860 (560, 1140) | 490 (340, 750). FeNO, ppb: 74.5 (54, 88) | 62 (35, 102). Exacerbations in past yr.: 4.5 (3, 7) | 5 (4, 8). mOCS dose, mg: 7.5 (5, 12.5) | 25 (12.5, 25). ACT score: 18 (14, 20) | 14.5 (12, 18)	Not reported
Chupp, 2017 [35]	Multinational	Phase IIIb RCT	556 (274–277)	Treatment: 49.8 (14.0). Placebo: 52.1 (12.9)	Data presented by placebo I treatment. M%: 36 | 46. BMI kg/m^2^: 27.9 (6.2) | 28.5 (6.6) (treatment). Ex‐smokers %: 27.0 | 26.0	Data presented by placebo | treatment. Pre‐BD FEV_1_% pred.: 55.2 (14.6) | 55.5 (14.4). BEC geometric mean (10^9^/L): 0.4 | 0.3. ACQ‐5 mean (SD): 2.2 (1.2) | 2.2 (1.1). Exacerbations in past yr., mean (SD): 2.7 (1.5) | 2.9 (1.9)	SC 100 mg/4 weeks
Crimi, 2020 [36]	Italy	Retrospective, single centre, real‐world study	31	52.4 (9.7)	M%: 42. BMI kg/m^2^, mean (SD): 26.68 (5.237). Current smokers %: 10.0	FEV_1_ (l), mean (SD): 2.15 (0.81). BEC, mean [median] [range] (SEM): 1219 [293–7180], [791] (273.8). IgE (IQR): 181 (88–355). Exacerbations/yr., median (IQR): 6 (4–12). mOCS *n* (%): 21 (67.7)	SC 100 mg/4 weeks for 12 month
Fong, 2021 [37]	UK	Retrospective, single centre real‐world study	240 (62–178)	Treatment: 52.0 (18.5), Comparator: 54.5 (25.0).	Data presented for treatment | comparator. M%: 33 | 30. BMI kg/m^2^, median (IQR): 28.95 (28.95) | 30.8 (10.8). Ever smokers%: 56.5 | 47.5	Data presented for treatment | comparator. FEV_1_%, mean (SD): 67.1 (20.8) | 76.8 (23.4). Max BEC median (IQR): 500 (400) | 200 (300). ACQ‐6 median (IQR): 2.7 (1.7) | 3 (1.8). FeNO median (IQR): 36 (52) | 16.9 (24.7). Exacerbation rate/yr., median (IQR): 4 (5) | 5 (3). mOCS %: 72.6 | 30.6	Route and dose not reported, administered every 4 weeks
Harvey, 2020 [9]	Australia	Prospective, multicentre, observational study	309	59.6 (50.0–68.3)	M%: 42, BMI kg/m^2^: 29.45 (25.30–34.46). Current smokers%: 0.6	Pre‐BD FEV_1_% pred. (*n* = 233): 56.9 ± 17.8. Blood eosinophils.μL^−1^ (*n* = 303): 590 (400–830). ACQ‐5 (*n* = 306): 3.4 (3.0–4.2). OCS course for exacerbation in past yr.: 295 (95.5). OCS courses in past yr.: 3.00 (2.00–6.00)	SC 100 mg/4 weeks
Kavanagh, 2020 [38]	UK	Retrospective, single centre, real‐world study	99	53.5 (13.2)	M%: 48. BMI kg/m^2^, mean (SD): 29.88 (5.76). Current smokers %: 4.5	FEV_1_% pred., mean (SD): 64.7 (21.3). BEC (cells×10^9^), median (IQR): 0.2 (0.1–0.4). FENO (ppb), median (IQR): 43.0 (27–73). ACQ‐6, mean (SD): 2.7 (1.3). Exacerbations/y, mean (SD): 4.0 (2.6). mOCS: 69%	SC 100 mg/4 weeks for ≥ 16 weeks
Pilette, 2022 [39]	Multinational	Prospective, single‐arm, observational cohort study	822	54.0 (13.6)	M%: 37. BMI kg/m^2^, mean (SD): 29.0 (7.24). Current/ex‐smoker%: 3.0/37.0	Asthma duration, y, mean (SD): 19.7 (15.7). BEC, cells/mL, geometric mean (SD log): 353 (1.2). OCS dose, mg/day, median (IQR): 10.0 (5.0–15.0). Exacerbations in past yr., mean (SD): 4.3 (4.1). Pre‐BD FEV_1_% pred.: 67.7 (21.1). ACQ‐5 score, mean (SD): 2.9 (1.3)	SC 100 mg/4 weeks
Reilly, 2023 [40]	UK	Retrospective, real‐world, single‐centre cohort	66	50.3 (14.7)	M%: 24. BMI kg/m^2^: 32.6 (7.3)	Median OCS exacerbations/y: 6.0 (5.0, 6.4). FEV1% pred.: 72.2 (22.3). ACQ‐6 (median): 3.3 (3.0, 3.9). BEC (median) nx10^9^/L: 0.42 (0.34, 0.51). FeNO ppb (median): 49.5 (38.7, 63.7)	SC 100 mg/4 weeks
Sasano, 2023 [41]	Japan	Prospective, observational cohort study	27	55.6 (13.4)	M%: 22. BMI kg/m^2^: 24.2 (4.7). Never/ex‐smoker %: 77.8/22.2	Asthma duration, y, mean (SD): 17.2 (11.6). Exacerbations/yr., median (IQR): 2.0 (0.0–3.0). FEV_1_% pred., mean (SD): 89.1 (24.8). BEC (cells/mL), median (IQR): 269.0 (161.0–486.0). FeNO (ppb), median (IQR): 25.0 (15.0–71.0). ACT score: 17.9 (5.3)	SC 100 mg/4 weeks
Thomas, 2021 [42]	Australia	Real‐world, observational study	309	59.6 (49.8, 68.2)	M%: 42. BMI, kg/m^2^, median (IQR): 29.52 (25.26, 34.42). Current/ex‐smoker %: 0.3/38.0	Asthma duration, yr., median (IQR): 27.5 (13.5, 46.1). % patients requiring OCS burst/s for exacerbations (past yr): 97.3. Pre‐BD FEV_1_% pred.: 57.0 (17.9). Post‐BD FEV_1_% pred.: 62.8 (19.0). ACT score, median (IQR): 11.0 (9.0, 15.0). BEC (cells/mL), median (IQR): 590.0 (400.0, 830.0). IgE (IU/mL), median (IQR): 141.5 (54.0, 461.5). FeNO (ppb), median (IQR): 35.0 (20.0, 61.0). OCS dose, (mg/day), median (IQR): 10.0 (5.0, 12.5)	Not reported but if following Australian guidelines: SC 100 mg/4 weeks
*Benralizumab*							
Al‐Ahmad, 2023 [43]	Kuwait	Retrospective, real‐world, single‐centre cohort	29	45.0 (12.8)	M%: 35. BMI kg/m^2^, mean (SD): 32.07 (8.22). Current smoker %: 21.6	*Data presented as mean* (*range*): ACT 13.3 (5–25). FEV_1_%: 58.3 (21–109). Total IgE (KU/L): 523.9 (10–5000). FeNO ppb: 26.4 (1–119). BEC: 499.9 (1–3460)	Not reported
Bilò, 2023 [33]	Italy	Multicentre real‐world study	49	57.0 (11.5)	M%: 33. BMI kg/m^2^: 26.2 (5.9). Current/ex‐smoker%: 4.1/30.6	Exacerbation rate/yr.: 3.5 (3.1). Pre‐BD FEV_1_% pred.: 66.0 (16.5). Post‐BD FEV_1_% pred.: 78.2 (19.1). Eosinophils/mm^3^: 628.9 (532.1). FeNO ppb: 34.2 (26.7). IgE values: 463.3 (930.7). ACT score: 16.9 (5.7)	Not reported
Kavanagh, 2020 [44]	UK	Retrospective, single centre, real‐world study	130	52.8 (14.0)	M%: 39. BMI kg/m^2^, mean (SD): 31.1 (7.1). Current/ex‐smoker%: 1.6/29.7	Exacerbation rate in yr. pre biologic, mean (SD): 4.9 (3.4). mOCS dose, mg/day, median (IQR): 10 (5–20). FEV_1_% pred., mean (SD): 63.8 (20.6). BEC, cells×10^9^/L, median (IQR): 0.2 (0.1–0.4). FENO, ppb, median (IQR): 45 (26–78). ACQ‐6 score, mean (SD): 2.9 (1.4)	SC 30 mg/4 weeks for 3 months, then 30 mg/8 weeks
Kroes, 2023^45^	Netherlands	*Post hoc* analysis of multicentre observational registry‐based real‐world study	192	58.0 (13.0)	M%: 48. BMI kg/m^2^, mean (SD): 28 (5.3). Former smoker: 47.9%	Exacerbation/yr., median (IQR): 3 (2–5). On mOCS %: 63.5. Prior biologic use%: 54.2. ACQ‐6 score BL, median (IQR): 2.2 (1.7–3.2). Pre‐BD FEV_1_% pred., mean (SD): 71.5 (22.1). FeNO (ppb), median (IQR): 41 (22–73). BEC (×10^9^cells L^−1^), median (IQR): 0.3 (0.1–0.6)	Not reported
Sandhu, 2023 [46]	Japan	Prospective observational study	31	54.3 (13.5)	M%: 29. BMI kg/m^2^: 24.2 (4.8). Never/ex‐smoker %: 71.0/29.0	FEV1% pred.: 83.2 (24.7). Exacerbations/yr., median (IQR): 3 (1–4). ACT score: 16.8 (5.6). Eosinophils (cells/uL): 80.0 (32.0–313.0). FeNO (ppb): 43 (15–74)	Not reported
Watanabe, 2021 [47]	Japan	Prospective, single‐arm, open‐label, observational, real‐world, study	24	55.0 (28–82)	M%: 38. BMI kg/m^2^: 23.1 (16.5, 30.3). No current smokers	FEV_1_% pred., median (range): 72.2 (32.6, 111.0). Blood eosinophil/μL: 228 (0, 5191). Total IgE (Iu/mL): 69 (5, 678). FeNO (ppb): 29.5 (5.0, 152.0). mOCS: 38%. ACQ: 2.4 (0.4, 5.4)	30 mg/4 weeks, then 30 mg/8 weekly
*Dupilumab*							
Domingo, 2022 [48]	Multinational	*Post hoc* analysis of Phase III RCT	210 (103–107)	Data presented as treatment | placebo for BL OCS dose < 10 mg/day; ≥ 10 mg/day. 55.4 (11.2) | 49.8 (13.6); 49.1 (12.8) | 51.2 (12.5)	Data presented as treatment | placebo for BL OCS dose < 10 mg/day; ≥ 10 mg/day. M%: 41 | 40; 39 | 39. BMI kg/m^2^, mean (SD): 28.0 (5.4) | 29.1 (6.1); 29.6 (6.3) | 30.1 (6.0). Former smokers %: 30.4 | 19.4; 17.5 | 14.1	Data presented as treatment | placebo for BL OCS dose < 10 mg/day; ≥ 10 mg/day. Pre‐BD FEV_1_ (L), mean (SD): 1.5 (0.6) | 1.6 (0.6); 1.6 (0.5) |1.6 (0.6). BEC (Giga/L), median (IQR): 0.3 (0.2–0.5) | 0.3 (0.2–0.5); 0.3 (0.2–0.5) | 0.2 (0.1–0.4). Total IgE, median (IU/mL), (IQR): 179.0 (96.0–389.0) | 143.0 (74.0–97.0); 201.0 (84.0–611.0) | 135.0 (36.0–307.0). FeNO (ppb), median (IQR): 27.0 (14.0–46.0) | 23.0 (15.0–52.0); 35.5 (16.5–51.0) | 30.0 (18.0–58.0). Exacerbations in past yr., mean (SD): 1.9 (1.9) | 1.7 (1.2); 2.1 (2.3) | 2.4 (2.6)	300 mg/2 weeks
Rabe, 2018 [49]	Multinational	RCT	210 (103–107)	51.3 (12.6)	M%: 40. BMI kg/m^2^, mean (SD): 29.34 (5.96). Former smokers %: 20.0	Pre‐BD FEV_1_% pred.: 52.2 (15.2). BEC (cells/mm^3^): 347 (307). FeNO (ppb): 37.6 (31.4). ACQ‐5: 2.5 (1.2). Exacerbations in past yr.: 2.1 (2.2). Daily oral glucocorticoid adjusted dose (mg/day): 11.3 (6.1)	SC 600 mg loading then 300 mg/2 weeks
Rabe, 2019 [50]	Multinational	*Post hoc* analysis of Phase III RCT	210 (103–107)	51.3 (12.6)	M%: 40. BMI kg/m^2^, mean (SD): 29.34 (5.96). Former smokers %: 20.0	Pre‐BD FEV_1_% pred.: 52.2 (15.2). BEC (cells/mm^3^): 347 (307). FeNO (ppb): 37.6 (31.4). ACQ‐5: 2.5 (1.2). Exacerbations in past yr.: 2.1 (2.2). Daily oral glucocorticoid adjusted dose (mg/day): 11.3 (6.1)	SC 600 mg loading then 300 mg/2 weeks
*Tezepelumab*							
Wechsler, 2022 [51]	Multinational	Phase III RCT	150 (74–76)	Treatment: 53.5 (12.1), Placebo: 53.4 (11.9)	Data presented by Treatment | Placebo. M%: 34 | 41. BMI kg/m^2^, mean (SD): 29.3 (6.7) | 29.4 (7.4)	Data presented by Treatment | Placebo: Pre‐BD FEV_1_% pred., mean (SD): 54.3 (18.1) | 53.3 (18.4). mOCS dose, mg, mean (SD): 11.1 (5.0)| 11.5 (6.0). BEC, cells/μL, mean (SD): 253 (203) | 232 (154). FeNO (ppb) mean (SD)^b^: 38.7 (40.8) | 42.4 (37.4). ACQ‐6 score, mean (SD): 2.48 (1.07) | 2.46 (1.03)	SC 210 mg/4 weeks

Abbreviations: CI, confidence interval; FEV_1_, forced expiratory volume in 1 s; ICS, inhaled corticosteroids; IV, intravenous dose; LSM‐TD, least squared mean treatment difference; OCS, oral corticosteroids; Q4W, treated every 4 weeks; Q8W, treated every 8 weeks; SC, subcutaneous dose; SD, standard deviation; SE, standard error.

^a^ Study reported switch/non‐switch data but classified as responder/non‐responder groups for the present review as the paper met eligibility criteria.

^b^ 68 and 69 participants had data at baseline.

The ability of inflammatory biomarkers to predict response was investigated by 17 studies, including BEC (11 studies) [9, 32, 34, 35, 36, 40, 44, 45, 47, 49, 51], FeNO (6 studies) [34, 44, 46, 47, 49, 50], BEC and FeNO combined (1 study) [50], T2 Phenotype (1 study) [33], Th17 cells (1 study) [46], Group 3 innate lymphoid cells (ILC3) (1 study) [46], periostin levels (1 study) [41], peripheral neutrophils (1 study) [41], and atopic status (4 studies) [9, 37, 44, 45]. Six studies assessed lung function parameters as predictors of response [33, 34, 37, 43, 44, 45]. 12 studies investigated clinical parameters for their predictive value, including exacerbation history (5 studies) [34, 37, 44, 45], OCS use/dose (10 studies) [34, 37, 39, 40, 42, 44, 45, 48], ICS dose (1 study) [45], prior biologic use (1 study) [45], number of respiratory medicines (1 study) [42], asthma control (7 studies) [34, 37, 38, 40, 42, 44, 45], QoL (1 study) [37], age of asthma onset (6 studies) [9, 37, 42, 43, 44, 45]. The presence of co‐morbidities as predictors of response was assessed in six studies, including allergic disease (1 study) [36], chronic rhinosinusitis (1 study) [45], non‐allergic rhinitis with eosinophilia syndrome (NARES) (1 study) [36], Nasal Polyps (NP) (5 studies) [33, 34, 36, 38, 45], bronchiectasis (2 studies) [36, 37], Gastroesophageal Reflux Disease (GERD) (2 studies) [33, 36], obesity (1 study) [36], osteoporosis (1 study) [33], dysfunctional breathing (1 study) [37], and mental health conditions (1 study) [37]. Eleven studies assessed socio‐demographic variables as predictors of response, including age (9 studies) [9, 33, 34, 36, 38, 40, 43, 44, 45], gender (6 studies) [9, 34, 36, 43, 44, 45], BMI (10 studies) [9, 33, 34, 36, 37, 40, 42, 43, 44, 45], smoking status (5 studies) [9, 36, 37, 44, 45] and geographic region (1 study) [33].

### 3.2 Risk of Bias in Included Studies

A summary of the risk of bias assessment for included studies is provided in Table 2. While most studies clearly defined the predictors of biologic response under investigation, about half lacked a diverse sample with varied clinical and demographic characteristics, leading to concerns about external validity. The primary reasons for negative ratings were a single‐centre real‐world design, small sample sizes (*n* < 30), or uneven gender distribution [32, 36, 37, 38, 41, 43, 44, 47]. None of the studies validated the response predictors identified in a training set of patients in a separate, validation patient set, limiting the generalizability of the findings to the broader severe asthma population. Only two studies [49, 51] evaluated the response predictors and outcomes in a blinded manner, putting the majority at risk of compromised internal validity. Blinding was not possible in 10 studies due to their retrospective real‐world [34, 36, 37, 38, 40, 43, 44] or *post hoc* design [45, 48, 50]. Although the original RCTs were conducted with blinding, *post hoc* analyses are particularly susceptible to publication bias, with significant results more likely to be reported. Notably, seven studies reported imprecise estimates of treatment effect, as indicated by wide confidence intervals [32, 34, 40, 41, 42, 46, 47], which limits the conclusiveness of their findings. This assessment did not consider reporting of *p*‐values due to their dependence on sample size.

**TABLE 2:** Risk of bias assessment for identified studies.

Reference, year	Clear definition/measurement of predictor	Population includes appropriate spectrum of patients	Predictor validated in a different group of patients	Predictor and outcome evaluated in a blinded fashion	Predictor and outcome evaluated in whole sample selected initially	Statistical methods used to assess the predictor clearly described	Precise estimate of treatment effect
*Mepolizumab*							
Bergantini, 2020 [32]	Y	N	N	N	Y	Y	N
Bilò, 2023 [33]	Y	Y	N	N	N	Y	Y
Caminati, 2023 [34]	N	Y	N	N^a^	N	N	N
Chupp, 2017 [35]	Y	Y	N	N	N	Y	Y
Crimi, 2020 [36]	Y	N	N	N^a^	Y	Y	Y
Fong, 2021 [37]	Y	N	N	N^a^	Y	Y	Y
Harvey, 2020 [9]	Y	Y	N	N	Y	Y	Y
Kavanagh, 2020 [38]	Y	N	N	N^a^	Y	Y	Y
Pilette, 2022 [39]	N	Y	N	N	N	Y	Y
Reilly, 2023 [40]	Y	N	N	N^a^	Y	Y	N
Sasano, 2023 [41]	N	N	N	N	N	N	N
Thomas, 2021 [42]	N	Y	N	N	N	Y	Y
*Benralizumab*							
Al‐Ahmad, 2023 [43]	Y	N	N	N^a^	Y	Y	Y
Bilò, 2023 [33]	Y	Y	N	N	N	Y	Y
Kavanagh, 2020 [44]	Y	N	N	N^a^	Y	Y	Y
Kroes, 2023 [45]	Y	Y	N	N^a^	Y	Y	Y
Sandhu, 2023 [46]	N	N	N	N	Y	Y	N
Watanabe, 2021 [47]	Y	N	N	N	Y	Y	N
*Dupilumab*							
Domingo, 2022 [48]	Y	Y	N	N^a^	Y	Y	Y
Rabe, 2018 [49]	Y	Y	N	Y	Y	Y	Y
Rabe, 2019 [50]	Y	Y	N	N^a^	Y	Y	Y
*Tezepelumab*							
Wechsler, 2022 [51]	Y	Y	N	Y	Y	Y	Y

*Note:* Green=Yes, Red=No.

Abbreviations: N, No; Y, Yes.

^a^ Retrospective study design or *post hoc* analysis and therefore could not evaluate predictor and outcome in blinded fashion.

### 3.3 Inflammatory Markers as Potential Biologic Response Predictors

The available evidence for inflammatory markers as predictors of biologic response is summarized in Table 3 (detailed in Table S2). For mepolizumab, ‘moderate’ quality evidence suggested that high BEC levels (≥ 300 and ≥ 500 cells/μL) are associated with greater improvement in asthma control defined by ACQ‐5 score [9, 35], compared to lower BEC levels (≥ 150 cells/μL), but not with improvement in the SGRQ score [35]. Similarly, ‘moderate’ quality indicated that high peak BEC (0.70 cells × 10^9^/L) in the year prior to benralizumab treatment is associated with a higher likelihood of super‐response [44], though evidence for high BEC levels predicting clinical response was inconsistent [44, 45]. The evidence was downgraded primarily due to heterogeneity in the measurement of predictor variables and response definitions across studies. For dupilumab and tezepelumab, ‘high’ quality evidence showed that high BEC levels (≥ 150 and ≥ 300 cells/μL) are associated with significantly greater reductions in exacerbations [49, 51], improvement in lung function [49, 50, 51] and a higher likelihood of OCS dose reduction [49, 51] compared to lower BEC levels (< 150 and < 300 cells/μL). Despite the limited number of studies, the evidence is based on large RCTs, lending greater confidence to the findings.

**TABLE 3:** Inflammatory biomarkers as predictors of response to biological therapies for severe asthma.

Biologic	Exacerbations	FEV_1_	Asthma control	(m)OCS use/dosage	Clinical response outcome
*Raised blood eosinophil counts*					
Mepolizumab	—	—	Higher BEC levels (≥ 300 and ≥ 500 cells/μL) are significantly associated with a greater likelihood of ACQ‐5 score improvement [9, 35] compared to lower BEC levels (≥ 150 cells/μL). However, when response is defined as SGRQ score improvement, BEC ≥ 500 cells/μL is associated with a lower likelihood of response compared to BEC ≥ 150 and ≥ 300 cells/μL [35]. ⨁⨁⨁◯^A,B,D,E^ MODERATE	—	It is unclear if higher BEC levels predict clinical response. Some studies suggest a significant association [34], with an optimal cut‐off of ≥ 580 cells/mm [3] for identifying partial responders [32]. In contrast, other studies report higher BEC (≥ 500/mm [3]) is not significantly associated with clinical response [36] but it is associated with negative response [40]. ⨁⨁◯◯ ^A,B,C,D^ **LOW**
Benralizumab	—	—	—	—	Higher peak BEC in the year prior to treatment is significantly associated with a greater likelihood of super‐response [44]. However, it is unclear if higher BEC levels predict clinical response. Some studies show a significant association, with an optimal cut‐off of 100/μL [47], while others report no significant association [44, 45]. ⨁⨁⨁◯ ^A,B,C,D^ **MODERATE**
Dupilumab	High BEC levels (≥ 300 cells/μL) are associated with a significantly greater reduction in exacerbation risk compared to BEC < 300 cells/μL. Lower BEC levels (< 150 cells/μL) are associated with a marginally greater reduction in exacerbation risk compared to ≥ 150 cells/μL, but both groups have smaller reductions than the ≥ 300 cells/μL group [49]. ⨁⨁⨁⨁^D,E^ **HIGH**	Higher BEC levels (≥ 300 cells/μL) are associated with significantly greater improvements in pre‐BD FEV_1_ compared to lower levels (< 300 cells/μL). BEC < 150 cells/μL are associated with marginally greater improvement than BEC > 150 cells/μL [49]. For post‐BD FEV_1_, higher BEC levels (≥ 150 and ≥ 300 cells/μL) are associated with greater improvements compared to lower BEC levels (< 150 and < 300 cells/μL), though improvements for the latter are statistically non‐significant [50]. ⨁⨁⨁⨁^C,D,E^ **HIGH**	—	Higher BEC levels (> 150 and > 300 cells/μL) are significantly associated with a greater reduction in OCS dose compared to lower BEC levels (BEC < 150 and < 300 cells/μL), with the largest reduction in the > 300 group. Higher BEC levels (> 300 cells/μL) also significantly increase the likelihood of achieving > 50% OCS reduction, reducing glucocorticoid dose to < 5 mg/day, and achieving steroid‐free status compared to lower BEC levels (< 300). In contrast, BEC > 150 cells/μL is associated with a lower likelihood of achieving glucocorticoid dose reduction to < 5 mg/day and steroid‐free status compared to BEC < 150 cells/μL, although some of the data are statistically not significant [49]. ⨁⨁⨁⨁^C,D,E^ **HIGH**	—
Tezepelumab	High BEC levels (≥ 150 and ≥ 300 cells/μL) are associated with a significant reduction in exacerbation rates, with the ≥ 300 group showing the greatest reduction. Low BEC levels (< 150 and < 300 cells/μL) are associated with increased exacerbation rates, though these changes were statistically not significant [51]. ⨁⨁⨁⨁^C,D,E^ **HIGH**	High BEC levels (≥ 150 and ≥ 300 cells/μL) are associated with significantly greater improvements in pre‐BD FEV_1_, with the largest improvement in the ≥ 300 group. In contrast, low BEC levels (< 150 and < 300 cells/μL) are associated with relatively modest, statistically non‐significant improvements [51]. ⨁⨁⨁⨁^C,D,E^ **HIGH**	Higher BEC (≥ 150 and ≥ 300 cells/μL) levels are not associated with significantly greater improvement in asthma control [51] ⨁⨁◯◯^C^ **LOW**	High BEC levels (≥ 150 and ≥ 300 cells/μL) are associated with a significantly higher likelihood of achieving clinically meaningful mOCS reductions, with the greatest likelihood in the ≥ 300 group. In contrast, low BEC levels (< 150 and < 300 cells/μL) show a reduced likelihood of OCS reduction, though the effect sizes are not statistically significant [51]. ⨁⨁⨁⨁ ^C,D,E^ **HIGH**	—
*FeNO*					
Mepolizumab	—	—	—	—	Raised FeNO is not significantly associated with clinical non‐response [34]. ⨁◯◯◯^A,C^ **VERY LOW**
Benralizumab	—	—	—	—	FeNO cut‐offs of 40 ppb and 44 ppb are associated with clinical response [47] and super‐response [46], respectively. However, one study suggests that elevated FeNO levels may not be significantly associated with clinical response [44]. ⨁⨁◯◯^A,C,D^ **LOW**
Dupilumab	FeNO levels > 25 to < 50 ppb are associated with a significantly greater reduction in exacerbation risk, compared to higher (> 50 ppb) and lower (< 25 ppb) levels, though the risk reductions for the latter are not significant [49] ⨁⨁⨁◯^C,D^ **MODERATE**	Low FeNO levels (< 25 ppb) are associated with significant improvements in pre‐ and post‐BD FEV_1_ [49, 50]. High FeNO levels (≥ 25 ppb) show inconsistent results, with one study indicating significant improvements in pre‐BD FEV_1_ but not post‐BD FEV_1_ [50], while another reports statistically insignificant increases in pre‐BD FEV_1_ [49]. ⨁⨁◯◯^B,C,D^ **LOW**	—	Elevated FeNO levels (> 25 to < 50 ppb and > 50 ppb) are significantly associated with oral glucocorticoid dose reduction, with levels between 25 and 50 ppb predicting a marginally greater reduction than levels above 50 ppb. FeNO level < 25 ppb is not significantly associated with glucocorticoid dose reduction [49]. ⨁⨁◯◯^C^ **LOW**	—
*Blood eosinophil counts* + *FeNO*					
Dupilumab	—	High eosinophil counts (≥ 150 cells/μL) plus low FeNO levels (< 25 ppb) are associated with significant improvements in pre‐ and post‐BD FEV_1_.When combined with high FeNO (≥ 25 ppb), high eosinophil counts are associated with improved post‐BD FEV_1_, but not pre‐BD FEV_1_. In contrast, combinations of lower eosinophil counts (< 150 cells/μL) with either low (< 25 ppb) or high (≥ 25 ppb) FeNO levels show inconsistent and generally non‐significant results [50]. ⨁⨁⨁◯^C,D^ MODERATE	—	—	—
*Raised periostin levels*					
Mepolizumab	—	—	—	—	A serum periostin level cut‐off of 92.5 ng/mL is associated with clinical response [41]. ⨁⨁⨁◯^C^ **MODERATE**
*Raised peripheral neutrophils*					
Mepolizumab	—	—	—	—	A peripheral neutrophil count cut‐off of 4035 cells/mL and neutrophil frequency of 61.6% (of white blood cells) are associated with clinical response [41]. ⨁⨁◯◯^C^ **LOW**
*T2 phenotype* ^a^					
Mepolizumab	—	T2 phenotype is not significantly associated with FEV_1_ improvement [33]. ⨁⨁◯◯^C^ **LOW**	T2 phenotype is not significantly associated with asthma control improvement [33]. ⨁⨁◯◯^C^ **LOW**	—	—
*Th17 cells*					
Benralizumab	—	—	—	—	High Th17 cell frequencies are associated with clinical response, with an optimal cut‐off of 4.57% for response, and 4.77% for super‐response [46]. ⨁⨁◯◯^C^ **LOW**
*ILC3*					
Benralizumab	—	—	—	—	Higher ILC3 levels are associated with clinical response, with an optimal cut‐off of 11.45% [46]. ⨁⨁◯◯^C^ **LOW**
*Atopic status*					
Mepolizumab	—	—	—	Atopic status is not significantly associated with asthma control improvement [9]. ⨁⨁◯◯^A^ **LOW**	Atopy is not significantly associated with clinical super‐response [37]. ⨁⨁◯◯^A^ **LOW**
Benralizumab	—	—	—	—	Non‐atopic and atopic phenotype are not significantly associated with clinical response [45] and super‐response [44] respectively. ⨁◯◯◯^A,C^ **VERY LOW**

*Note:* GRADE Working Group grades of evidence: *High certainty*: High confidence that the true effect lies close to that of the estimate of the effect. *Moderate certainty*: Moderate confidence in the effect estimat; the true effect is likely to be close to the estimate of the effect, but there is a possibility that it is substantially different. *Low certainty*: Limited confidence in the effect estimat; the true effect may be substantially different from the estimate of the effect. *Very low certainty*: Very little confidence in the effect estimat; the true effect is likely to be substantially different from the estimate of effect. Explanations: The following factors reduce the certainty of evidence: A: risk of bias; B: inconsistency of results; C: imprecision. The following factors increase the certainty of evidence: D: large magnitude of effect; E: dose–response gradient. Green = investigated variable predicts positive biologic response, blue = no association between investigated variable and biologic response.

Abbreviations: ACQ, Asthma Control Questionnaire; BEC, blood eosinophil counts; FeNO, fractional exhaled nitric oxide; FEV_1_ , forced expiratory volume in 1 s; ILC3, Group 3 innate lymphoid cells; mOCS, maintenance oral corticosteroids.

^a^ T2 phenotype definitions included ‘IgE > 150 and/or Eos > 300 and/or FeNO > 25’; ‘IgE > 150 and/or Eos > 150 and/or FeNO > 25’.

**FIGURE 2:**
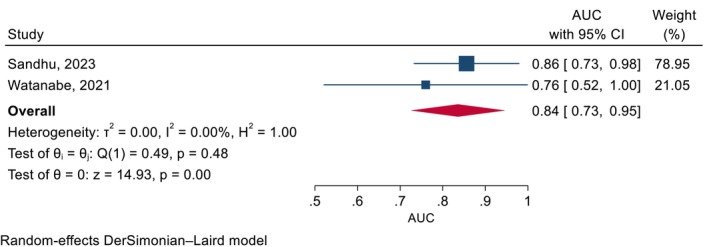
Elevated FeNO levels as a predictor of clinical response to Benralizumab. Elevated FeNO levels cut‐offs of 40–44 ppb. AUC, area under curve; CI, confidence intervals.

Some studies suggested that high FeNO levels (> 40 ppb) were associated with clinical response [47] and super‐response [46] to benralizumab, while others reported no significant predictive value [44]. The meta‐analysis revealed that elevated FeNO levels in the range of 40–44 ppb have good discriminatory ability for identifying clinical responders versus non‐responders to benralizumab (Figure 2). In contrast, evidence for FeNO as a predictor of mepolizumab response was limited and of ‘very low’ quality; a small observational study suggested that elevated FeNO levels were not associated with clinical non‐response but did not provide comparisons between different cut‐off values [34].

FeNO levels between 25 and 50 ppb were associated with a greater reduction in exacerbation risk and OCS dose following dupilumab, compared to both higher (> 50 ppb) and lower (< 25 ppb) levels [49]. FeNO < 25 ppb was associated with lung function improvement, though post‐BD FEV_1_ improvements did not reach the Minimal Important Difference (MID) of 0.2L [49, 50]. A *post hoc* analysis of a large RCT showed that combining high BEC (≥ 150 cells/μL) with low FeNO levels (< 25 ppb) predicts clinically significant improvements in pre‐ and post‐BD FEV_1_, surpassing the MID [50]. However, the evidence quality was downgraded to ‘moderate’ due to imprecision indicated by wide confidence intervals.

There was predominantly ‘low’ quality evidence that biomarkers such as high periostin levels, peripheral neutrophils [41], Th17 cells, and ILC3 [46] are associated with anti‐IL5/5Rα response. This evidence was downgraded due to imprecision, arising from the small sample size of the included observational studies.

### 3.4 Lung Function Parameters as Potential Biologic Response Predictors

Lung function and airflow obstruction parameters (such as FEV_1_ < 60% and FEV_1_/FVC < LLN) were not significantly associated with a reduction in exacerbations or clinical (non‐) response following mepolizumab [34, 37] and benralizumab treatment [43, 44, 45] (Table 4; detailed synthesis in Table S3). The quality of evidence was rated ‘very low’ due to a serious risk of bias, stemming from heterogeneity in response definitions and the lack of comparison between different thresholds for the investigated lung function parameters. The small sample sizes of the included real‐world studies and imprecise effect estimates further reduced the certainty of evidence.

**TABLE 4:** Lung function parameters as predictors of response to biological therapies for severe asthma.

Biologic	Exacerbations	Clinical response outcome
*Normal FEV*_*1*_		
Mepolizumab	—	FEV_1_ variables (such as FEV_1_% predicted and FEV_1_ L) are not significantly associated with super‐response or non‐response [34, 37]. ⨁◯◯◯^A,C^ **VERY LOW**
Benralizumab	—	FEV_1_ variables (such as FEV_1_ < 60%, FEV_1_% predicted, and pre‐BD FEV_1_% predicted) are not significantly associated with clinical response or super‐response [43, 44, 45]. ⨁◯◯◯^A,C^ **VERY LOW**
*Normal FEV*_*1*_ */FVC< LLN*		
Mepolizumab	FEV_1_/FVC< LLN is not significantly associated with a reduction in exacerbations [33] ⨁◯◯◯^A,C^ **VERY LOW**	—
Benralizumab	FEV_1_/FVC< LLN is not significantly associated with a reduction in exacerbations [33] ⨁◯◯◯^A,C^ **VERY LOW**	—

*Note:* GRADE Working Group grades of evidence: *High certainty*: High confidence that the true effect lies close to that of the estimate of the effect. *Moderate certainty*: Moderate confidence in the effect estimate: the true effect is likely to be close to the estimate of the effect, but there is a possibility that it is substantially different. *Low certainty*: Limited confidence in the effect estimate: the true effect may be substantially different from the estimate of the effect. *Very low certainty*: Very little confidence in the effect estimate: the true effect is likely to be substantially different from the estimate of effect. Explanations: The following factors reduce the certainty of evidence: A: risk of bias; B: inconsistency of results; C: imprecision. The following factors increase the certainty of evidence: D: large magnitude of effect; E: dose–response gradient. Blue = no association between investigated variable and biologic response.

Abbreviations: ACQ, Asthma Control Questionnaire; FEV_1_ , forced expiratory volume in 1 s; FVC, forced vital capacity; LLN, lower limit of normal FEV1/FVC; pre‐BD, pre‐bronchodilator.

### 3.5 Clinical Parameters as Potential Biologic Response Predictors

The quality of evidence for the biologic response prediction value of clinical parameters is presented in (Table 5; detailed synthesis in Table S4). For Mepolizumab, a high baseline exacerbation rate was a significant predictor of super‐response [37] but the evidence quality was rated ‘low’ due to risk of bias and imprecision. There was ‘high’ quality evidence that lack of OCS use or low OCS dose (< 10 mg/day) at baseline is associated with a reduced risk of exacerbations [39], while baseline OCS use reduced the likelihood of achieving OCS‐free status [42]. For dupilumab, a low OCS dose (< 10 mg/day) was significantly associated with a greater reduction in exacerbation rates, greater improvement in pre‐BD FEV_1_, and a higher likelihood of achieving steroid‐free status compared to high OCS dose (≥ 10 mg/day) [48]. This evidence was upgraded to ‘high’ quality due to the clinical significance of the effect estimates and a clear dose–response gradient. Biologic naïve patients were more likely to achieve benralizumab response [45], with the evidence upgraded to ‘high’ certainty due to the large magnitude of effect. Adult‐onset asthma was associated with greater asthma control improvement [9] and an increased likelihood of achieving OCS‐free status [42] following mepolizumab, though evidence for the latter was rated ‘very low’ due to lack of comparative data on age cut‐offs. On the other hand, there was ‘moderate’ quality evidence that childhood‐onset asthma is associated with benralizumab response [43].

**TABLE 5:** Clinical parameters as predictors of response to biological therapies for severe asthma.

Biologic	Exacerbations	FEV_1_	Asthma control	(m)OCS use/dosage	Clinical response outcome
*Worse exacerbation history*					
Mepolizumab	—	—	—	—	A higher exacerbation rate at baseline is predictive of super‐response [37]. Exacerbation history is not associated with non‐response [34]. ⨁⨁◯◯^A,C,D^ **LOW**
Benralizumab	—	—	—	—	Exacerbation history is not associated with clinical response [44, 45] or super‐response [44]. ⨁◯◯◯^A,C^ **VERY LOW**
*OCS use/dose*					
Mepolizumab	Not being on mOCS or being on lower doses (< 10 mg/day) is significantly associated with a reduced risk of exacerbations compared to being on mOCS or higher doses (≥ 10 mg/day) [39]. ⨁⨁⨁⨁^D,E^ **HIGH**	—	—	Baseline mOCS use significantly decreases the likelihood of achieving OCS‐free status after 6 months of treatment [42]. ⨁⨁⨁⨁^D^ **HIGH**	Higher OCS daily dose is associated with a higher likelihood of non‐response [34]. Conversely, mOCS use is not significantly associated with clinical response [37, 40]. ⨁◯◯◯^A,C^ **VERY LOW**
Benralizumab	—	—	—	—	OCS use is associated with reduced likelihood of super‐response, but when combined with other variables, it becomes predictive [44]. In contrast, a higher OCS dose at 3 months significantly lowers the likelihood of a positive clinical response [45]. ⨁◯◯◯^A,C^ **VERY LOW**
Dupilumab	A low OCS dose (< 10 mg/day) is significantly associated with a greater reduction in exacerbations compared to a high OCS dose (≥ 10 mg/day) [48]. ⨁⨁⨁⨁^D,E^ **HIGH**	A low OCS dose (< 10 mg/day) is significantly associated with a greater improvement in post‐BD FEV_1_ compared to a high OCS dose (≥ 10 mg/day). In contrast a high OCS dose (≥ 10 mg/day) is significantly associated with a greater improvement in pre‐BD FEV_1_ compared to low dose OCS (< 10 mg/day) [48]. ⨁⨁⨁⨁^C,D,E^ **HIGH**	—	A low OCS dose (< 10 mg/day) is significantly associated with higher likelihood of achieving steroid‐free status compared to a high OCS dose (≥ 10 mg/day) [48]. ⨁⨁⨁⨁^D,E^ **HIGH**	—
*Increased ICS dose*					
Benralizumab	—	—	—	—	Higher ICS dose at baseline is not associated with clinical response [45]. ⨁⨁⨁◯^A^ **MODERATE**
*Biologic naïve*					
Benralizumab	—	—	—	—	Being biologic naïve is associated with clinical response [45]. ⨁⨁⨁⨁^D^ **HIGH**
*Increased number of respiratory medicines*					
Mepolizumab	—	—	—	Higher number of respiratory medications at baseline is not significantly associated with achieving steroid‐free status [42] ⨁⨁◯◯^C^ **LOW**	—
*Better asthma control*					
Mepolizumab	—	—	—	Better asthma control is associated with increased likelihood of achieving OCS‐free status after 6 months of treatment [42]. ⨁⨁⨁⨁ **HIGH**	Better asthma control is significantly associated with clinical response and super‐response [37, 38], but not with non‐response [34]. ⨁⨁⨁◯^A,C,D^ **MODERATE**
Benralizumab	—	—	—	—	Better asthma control is not significantly associated with clinical response [44, 45] or super‐response [44]. ⨁◯◯◯^A,C^ **VERY LOW**
					Better asthma control 3 months post treatment significantly increases the likelihood of achieving clinical response [45]. ⨁⨁⨁⨁^A,D^ **HIGH**
*Better quality of life*					
Mepolizumab	—	—	—	—	QoL is not significantly associated with clinical response [37]. ⨁⨁◯◯^A^ **LOW**
*Adult‐onset asthma*					
Mepolizumab	—	—	Later age of asthma onset (≥ 40 years) is associated with greater improvement in asthma control, but the MCID is not achieved [9]. ⨁⨁⨁⨁ **HIGH**	Later age of asthma onset is associated with a marginally increased likelihood of achieving OCS‐free status after 6 months, but this is not clinically meaningful [42]. ⨁◯◯◯^A,C^ **VERY LOW**	Adult‐onset asthma is not significantly associated with clinical response [37]. ⨁⨁◯◯^A^ **LOW**
Benralizumab	—	—	—	—	Adult‐onset asthma is not significantly associated with clinical response [44, 45] or super‐response [44]. ⨁◯◯◯^A,C^ **VERY LOW**
*Childhood‐onset asthma*					
Benralizumab	—	—	—	—	Childhood‐onset asthma is associated with clinical response [43]. ⨁⨁⨁◯^C^ **MODERATE**

*Note:* GRADE Working Group grades of evidence: *High certainty*: High confidence that the true effect lies close to that of the estimate of the effect. *Moderate certainty*: Moderate confidence in the effect estimate: the true effect is likely to be close to the estimate of the effect, but there is a possibility that it is substantially different. *Low certainty*: Limited confidence in the effect estimate: the true effect may be substantially different from the estimate of the effect. *Very low certainty*: Very little confidence in the effect estimate: the true effect is likely to be substantially different from the estimate of effect. Explanations: The following factors reduce the certainty of evidence: A: risk of bias; B: inconsistency of results; C: imprecision. The following factors increase the certainty of evidence: D: large magnitude of effect; E: dose–response gradient. Green = investigated variable predicts positive biologic response, red = investigated variable predicts negative biologic response, blue = no association between investigated variable and biologic response.

Abbreviations: ACQ, Asthma Control Questionnaire; FEV_1_ , forced expiratory volume in 1 s; MCID, minimally clinically important difference; mOCS, maintenance oral corticosteroids; QoL, quality of life.

**FIGURE 3:**
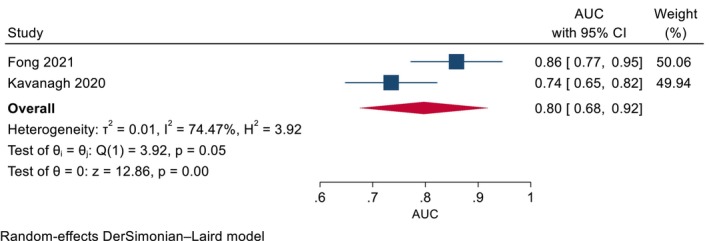
Better asthma control at baseline as a predictor of clinical response to mepolizumab. Asthma control measured using the Asthma Control Questionnaire. AUC, area under curve; CI, confidence intervals.

Better asthma control at baseline was associated with a higher likelihood of achieving OCS‐free status [42], as well as clinical response and super‐response [37, 38] following mepolizumab treatment, with ‘high’ and ‘moderate’ quality evidence, respectively. The forest plot in Figure 3 shows that better asthma control at baseline, defined as a low Asthma Control Questionnaire score, may be able to distinguish responders versus non‐responders to mepolizumab. However, the high heterogeneity between the included studies suggests that this relationship may not be consistent across populations. While no association was found between baseline asthma control and response to benralizumab, a *post hoc* analysis of a large real‐world study showed that better asthma control at 3 months can predict long‐term clinical response [45].

### 3.6 Co‐morbidities as Potential Biologic Response Predictors

The quality of evidence for co‐morbidities as predictors of response to biologics for severe asthma is summarised in Table 6 (detailed in Table S5). NP was significantly associated with clinical response and super‐response to mepolizumab [38], but the evidence quality was rated ‘very low’ owing to heterogeneity in response definitions and the small sample size of the included studies. NP in patients treated with benralizumab was associated with a reduced likelihood of response, but the ‘low’ quality evidence was based on a small real‐world cohort [33]. Evidence for the predictive value of other co‐morbidities, including allergy [36], chronic rhinosinusitis [45], NARES [36], Bronchiectasis [36, 37], GERD [33, 36], obesity [36], osteoporosis [33], dysfunctional breathing, and mental health disorders [37], was inconclusive and of ‘low’ to ‘very low’ quality. This was primarily due to the lack of comparative data for patients with and without these co‐morbidities, as well as imprecise effect estimates indicated by wide confidence intervals.

**TABLE 6:** Co‐morbidities as predictors of response to biological therapies for severe asthma.

Biologic	Exacerbations	Asthma control	Clinical response outcome
*Allergic disease*			
Mepolizumab	—	—	Allergy is not significantly associated with clinical response [36]. ⨁◯◯◯^A,C^ **VERY LOW**
*Chronic rhinosinusitis*			
Benralizumab	—	—	Chronic rhinosinusitis is not a significant predictor of clinical response [45]. ⨁◯◯◯^A,C^ **VERY LOW**
*NARES*			
Mepolizumab	—	—	NARES is not significantly associated with clinical response [36]. ⨁◯◯◯^A,C^ **VERY LOW**
*Nasal Polyps*			
Mepolizumab	—	NP is not significantly associated with improvement in asthma control [33]. ⨁◯◯◯^A,C^ **VERY LOW**	NP is significantly associated with clinical response and super‐response [38], but not with non‐response [34]. ⨁◯◯◯^A,C^ **VERY LOW**
Benralizumab	—	NP is associated with reduced likelihood of asthma control improvement [33]. ⨁⨁◯◯^A,C^ **LOW**	NP is not a significant predictor of clinical (super) response [44, 45]. ⨁◯◯◯^A,C^ **VERY LOW**
*Bronchiectasis*			
Mepolizumab	—	—	Bronchiectasis is not significantly associated with clinical response [36, 37]. ⨁◯◯◯^A,C^ **VERY LOW**
*GERD*			
Mepolizumab	GERD is not significantly associated with reduction in exacerbations [33]. ⨁◯◯◯^A,C^ **VERY LOW**	GERD is not significantly associated with asthma control improvement [33]. ⨁◯◯◯^A,C^ **VERY LOW**	GERD is not significantly associated with clinical response [36]. ⨁◯◯◯^A,C^ **VERY LOW**
Benralizumab	GERD is not significantly associated with reduction in exacerbations [33]. ⨁◯◯◯^A,C^ **VERY LOW**	GERD is not significantly associated with asthma control improvement [33]. ⨁◯◯◯^A,C^ **VERY LOW**	—
*Obesity*			
Mepolizumab	—	—	Obesity is not significantly associated with clinical response [36]. ⨁◯◯◯^A,C^ **VERY LOW**
*Osteoporosis*			
Mepolizumab	—	Osteoporosis is not significantly associated with asthma control improvement [33]. ⨁◯◯◯^A,C^ **VERY LOW**	—
Benralizumab	—	Osteoporosis is associated with a lower likelihood of achieving asthma control improvement, but it is unclear if this is clinically meaningful [33]. ⨁⨁◯◯^A,C^ **LOW**	—
*Dysfunctional breathing*			
Mepolizumab	—	—	Dysfunctional breathing is not significantly associated with clinical response [37]. ⨁⨁◯◯^A^ **LOW**
*Mental health conditions*			
Mepolizumab	—	—	Depression and anxiety are not significantly associated with clinical response [37]. ⨁⨁◯◯^A^ **LOW**

*Note:* GRADE Working Group grades of evidence: *High certainty*: High confidence that the true effect lies close to that of the estimate of the effect. *Moderate certainty*: Moderate confidence in the effect estimate: the true effect is likely to be close to the estimate of the effect, but there is a possibility that it is substantially different. *Low certainty*: Limited confidence in the effect estimate: the true effect may be substantially different from the estimate of the effect. *Very low certainty*: Very little confidence in the effect estimate: the true effect is likely to be substantially different from the estimate of effect. Explanations: The following factors reduce the certainty of evidence: A: risk of bias; B: inconsistency of results; C: imprecision. The following factors increase the certainty of evidence: D: large magnitude of effect; E: dose–response gradient. Green = investigated variable predicts positive biologic response, red = investigated variable predicts negative biologic response, blue = no association between investigated variable and biologic response.

Abbreviations: ACQ, Asthma Control Questionnaire; FEV_1_ , Forced expiratory volume in 1 s; GERD, Gastroesophageal reflux disease; mOCS, maintenance oral corticosteroids; NARES, nonallergic rhinitis with eosinophilia syndrome; NP, nasal polyposis.

### 3.7 Socio‐demographic Characteristics as Potential Biologic Response Predictors

A summary of the quality of evidence for socio‐demographic characteristics as predictors of biologic response is presented in Table 7 (detailed synthesis in Table S6). There was ‘moderate’ to ‘low’ quality evidence that a higher BMI (> 30 kg/m^2^) is associated with a higher likelihood of experiencing more exacerbations [33], reduced asthma control improvement [9], and a lower likelihood of achieving steroid‐free status [42] following mepolizumab treatment. Regarding gender, a single large observational study provided ‘low’ quality evidence that male participants are less likely to benefit from mepolizumab in terms of asthma control improvement [9]. Other investigated variables, such as age and smoking status, were not associated with anti‐IL5/5Rα response, with several studies lacking comparative data on clearly defined age cut‐offs [9, 43, 44, 45] and smokers versus non‐smokers [36, 37].

**TABLE 7:** Socio‐demographic characteristics as predictors of response to biological therapies for severe asthma.

Biologic	Exacerbations	FEV_1_	Asthma control	(m)OCS use/dosage	Clinical response outcome
*Age*					
Mepolizumab	—	Older age (> 50 years) is not significantly associated with FEV_1_ improvement [33]. ⨁◯◯◯^A,C^ **VERY LOW**	Age is not significantly associated with improvement in asthma control [9] (unclear whether this is older or younger age). ⨁⨁◯◯^A^ **LOW**	—	Younger age is a borderline predictor of clinical non‐response [34]. The relationship between older age and clinical response is inconsistent; some studies suggest older age is associated with positive response [38], while others report no significant association with response [36, 40] or super‐response [38]. ⨁◯◯◯^A,B,C^ **VERY LOW**
Benralizumab	—	Older age (> 50 years) is not significantly associated with FEV_1_ improvement [33]. ⨁◯◯◯^A,C^ **VERY LOW**	—	—	Age is not significantly associated with clinical response. However, age thresholds were not defined in any of the included studies [43, 44, 45]. ⨁◯◯◯^A,C^ **VERY LOW**
*Gender*					
Mepolizumab	—	—	Male gender is associated with reduced asthma control improvement [9]. ⨁⨁◯◯^A^ **LOW**	—	Female gender is not significantly associated with negative [34] or positive clinical response [36]. ⨁◯◯◯^A,C^ **VERY LOW**
Benralizumab	—	—	—	—	Female gender is not significantly associated with negative response [43], or positive response [44]. Male gender is not significantly associated with clinical response [45]. ⨁◯◯◯^A,C^ **VERY LOW**
*High BMI*					
Mepolizumab	BMI > 30 kg/m^2^ is associated with higher likelihood of experiencing more exacerbations [33]. ⨁⨁⨁◯^A^ **MODERATE**	—	BMI ≥ 30 kg/m^2^ is associated with reduced asthma control improvement [9]. ⨁⨁◯◯^A^ **LOW**	Higher BMI is associated with decreased likelihood of achieving steroid‐free status after 6 months of treatment [42]. ⨁⨁⨁◯^A^ **MODERATE**	Higher BMI is not significantly associated with clinical response [34, 36, 37, 40]. ⨁◯◯◯^A,C^ **VERY LOW**
Benralizumab	—	—	—	—	Higher BMI is not significantly associated with negative response [43], positive response or super‐response [44, 45]. ⨁◯◯◯^A,C^ **VERY LOW**
*Smoking status*					
Mepolizumab	—	—	Smoking status (ex−/current or never) is not associated with an improvement in asthma control [9]. ⨁⨁◯◯^A^ **LOW**	—	Smoking is not significantly associated with clinical response [36, 37]. ⨁◯◯◯ ^A,C^ **VERY LOW**
Benralizumab	—	—	—		Being an ex‐smoker is not associated with clinical response [45]. ⨁⨁◯◯^C^ **LOW**
*Geographic region*					
Mepolizumab	—	—	Rural residence is not associated with improvement in asthma control [33]. ⨁⨁◯◯^C^ **LOW**	—	—
Benralizumab	—	—	Rural residence is not associated with improvement in asthma control [33]. ⨁⨁◯◯^C^ **LOW**	—	—

*Note:* GRADE Working Group grades of evidence: *High certainty*: High confidence that the true effect lies close to that of the estimate of the effect. *Moderate certainty*: Moderate confidence in the effect estimate: the true effect is likely to be close to the estimate of the effect, but there is a possibility that it is substantially different. *Low certainty*: Limited confidence in the effect estimate: the true effect may be substantially different from the estimate of the effect. *Very low certainty*: Very little confidence in the effect estimate: the true effect is likely to be substantially different from the estimate of effect. Explanations: The following factors reduce the certainty of evidence: A: risk of bias; B: inconsistency of results; C: imprecision. The following factors increase the certainty of evidence: D: large magnitude of effect; E: dose–response gradient. Green = investigated variable predicts positive biologic response, red = investigated variable predicts negative biologic response, blue = no association between investigated variable and biologic response.

Abbreviations: BMI, Body Mass Index; FEV_1_ , Forced expiratory volume in 1 s; mOCS, maintenance oral corticosteroids.

## 4 Discussion

This systematic review is the first comprehensive synthesis of evidence on predictors of response to anti‐IL5/5Rα, −4Rα, and anti‐TSLP therapies for severe asthma. We found ‘moderate’ to ‘high’ quality evidence that high BEC (≥ 300 cells/μL) reliably predicts response to mepolizumab, dupilumab, and tezepelumab. Elevated FeNO levels (> 40 ppb) predicted benralizumab response, while FeNO levels between 25 and 50 ppb were linked to reduced exacerbations and OCS dose after dupilumab treatment. ‘High’ quality evidence showed that being OCS naïve or lower baseline OCS dose (< 10 mg/day) is associated with better outcomes following mepolizumab and dupilumab. Additionally, better asthma control at baseline and post 3 months were significant predictors of mepolizumab and benralizumab response, respectively. However, evidence for other variables was limited, downgraded primarily due to the heterogeneous measurement and reporting of response definitions. This highlights the urgent need for standardized, universally accepted definitions of response in studies of targeted therapies for severe asthma. Additionally, no evidence was found for biologics targeting the non‐T2 pathway or the pediatric severe asthma population.

Our finding that elevated BEC levels can predict response to anti‐IL‐5/5R, anti‐IL‐4, and anti‐TSLP therapies aligns with GINA guidance, which recommends using raised BEC levels for biologic initiation assessment [16]. Several large RCTs and *post hoc* analyses that informed the regulatory approval of T2 biologics [52, 53, 54, 55, 56, 57, 58] and reported on the response predictive value of raised BEC levels [59, 60] were not eligible for this review due to their inclusion of patients with moderate‐to‐severe asthma or lack of subgroup analysis. Consequently, it was not possible to conduct a meta‐analysis to quantitatively delineate the BEC cut‐offs for predicting response in the true severe asthma population. Nonetheless, the real‐world studies comprising most of our evidence base represented a heterogeneous clinic population, where treatment response can be influenced by unique non‐pathophysiologic factors that are typically absent in RCTs. However, our conclusions were limited by the small sample size of the eligible studies, highlighting the need for larger real‐world studies to better understand the traits influencing biologic response in the severe asthma subgroup [61, 62].

The practical application of biologic response predictors requires careful consideration of feasibility. This review found that raised FeNO levels (> 40 ppb) can identify responders to benralizumab, and levels between 25 and 50 ppb are linked to greater benefit with dupilumab. However, FeNO remains less commonly used in practice compared to biomarkers like BEC, primarily due to the ease of measuring BEC, while FeNO testing may be limited by equipment availability in certain clinical settings. Feasibility becomes even more critical when considering exploratory predictors, such as genetic polymorphisms in the IL‐4, 5, and 13 pathways [63]. While genetic testing holds potential for biologic response prediction, it introduces challenges related to cost, integration into clinical workflows, and real‐time data access to guide treatment decisions. Addressing these feasibility concerns is key to ensuring the rapid adoption of emerging treatment response predictors into practice.

The ‘high’ quality evidence supporting OCS naïveté and low OCS dose as predictors of mepolizumab and dupilumab response aligns with the literature, which shows that OCS use is associated with poor response to anti‐IL5 [12], while OCS naïveté or low baseline OCS dose predicts remission [64]. However, the predominantly ‘low’ quality evidence for the response predictive value of other variables across clinical, lung function, sociodemographic, and co‐morbidity domains contrasts with the wider literature. For example, although our review found limited ‘very low’ quality evidence for NP as a predictor of mepolizumab response, *post hoc* analyses [65] suggest that NP is associated with greater reduction in exacerbations, potentially serving as a surrogate T2 marker. This disparity stemmed from studies such as the latter recruiting moderate‐to‐severe asthma patients, with those included in our review downgraded due to inconsistent definitions and measurements of predictors (e.g., patient‐reported vs. clinically diagnosed NP) and lack of comparison data for patients with versus without the predictor (e.g., effect size reported for NP group only), or above versus below predictor thresholds. Standardizing the definition, measurement, and reporting of predictor variables in future studies is critical to enable data pooling for meta‐analysis and drawing more definitive conclusions.

Heterogeneity in response definitions, particularly among studies using clinical response criteria, was a major factor in downgrading the quality of evidence and hindering data pooling for meta‐analysis. Response criteria varied across studies in terms of measurement (e.g., exacerbation reduction measured by hospitalisations vs. OCS bursts), definition (e.g., > 50% vs. 80% exacerbation reduction), and outcome combinations (e.g., clinical, inflammatory, and patient‐reported vs. clinical plus patient‐reported only). This inconsistency led to cases where predictors of response in one study did not hold true in others with differing definitions. Additionally, bias was introduced when the predictor variable was part of a clinical response definition, thus increasing the likelihood of meeting that definition. There was also variability in the directionality of effect, with some studies assessing a variable's ability to predict response, while others focused on its role in predicting non‐response. To address these issues, there is a clear need to implement a universal response definition, such as CONFIRM [66], and further research to unravel the relationships between predictors and biologic response, alongside the development of multivariate prediction tools [67].

We found limited evidence for the response predictive value of variables such as periostin, yet T2 biomarkers are often used in practice to define eligibility for biological therapy. This underscores the need for robust studies to identify reliable predictors beyond traditional markers that influence individual patient response. The 3TR Asthma Biologics Cohort study [68] is addressing this gap by using advanced multi‐omics methods and repeated bio‐sampling from multiple compartments to identify biomarkers that can predict (non‐) response. To facilitate a step change, funding bodies such as the EU/IHI and NIH should prioritize and support more controlled interventional trials focused on emerging biomarkers.

Although six studies in this review indicated eligibility for patients with severe asthma aged ≥ 12 years [9, 35, 36, 48, 49, 50], most participants were adults. A previous review [69] reported on clinically available biomarkers and their ability to predict T2 biologic response in children, but it primarily focused on moderate‐to‐severe asthma, highlighting a gap in research specifically targeting the paediatric severe asthma population. Additionally, our review identified a lack of data for non‐T2 populations. Given the observed benefits of anti‐TSLP therapy in patients with non‐T2 characteristics [53, 70, 71, 72], further investigation into response predictors for the non‐atopic population is urgently needed.

This study excluded predictors of omalizumab response to prevent repetition, as a comprehensive review has already been conducted and published on this [23]. That analysis identified baseline BEC and total serum IgE levels as reliable predictors of omalizumab response in patients with allergic asthma [23].

### 4.1 Strengths and Limitations

This systematic review has several strengths. A highly sensitive search strategy was developed and used to search four bibliographic databases and two clinical trial registries. Members of the 3TR Respiratory Work Package were also consulted to ensure no relevant studies were missed. Another strength was the inclusion of only studies focused on severe asthma as defined by the ATS/ERS guidelines [6]. Although this approach excluded several large RCTs involving participants with moderate to severe asthma, it preserved the integrity of the synthesis and ensured comparability across the included studies. Further, the outcomes were selected based on the multi‐national COMSA consensus study, ensuring their relevance to key stakeholders, including patients [25, 26]. A rigorous appraisal strategy was employed, including risk of bias assessment using the CASP CPR [28] checklist and certainty of evidence rating using the modified GRADE framework [29, 30, 31]. This standardised approach ensured a robust and transparent assessment of the included studies and minimised bias.

A limitation of this review was the inconsistency in measurement of response across the included studies, hindering the ability to perform meta‐analysis. To address this, future studies should use standardized definitions of response, such as CONFiRM [66]. The review was limited to studies published in English; however, we screened studies cited in guidelines, reviews, and references of identified records to ensure no relevant studies were missed. The exclusion of studies reporting predictors of omalizumab response meant that it was not possible to draw comparisons with data for other biologics. Nonetheless, given that a review focused on omalizumab response predictors already exists [23], this exclusion avoids redundancy. The evidence base for tezepelumb was limited to one eligible study [51], primarily because the synthesis was conducted shortly after the biologic was introduced into clinical practice. A total of 17 studies reporting on response predictors for pooled datasets of multiple biologics were excluded, as we did not receive data specific to each biologic despite requests to the authors. Amalgamating these data was not appropriate due to the distinct mechanisms of action of the different biologics.

### 4.2 Implications for Research and Practice

This systematic review aimed to inform healthcare providers and regulators about the available evidence for predictors of response to biologics for severe asthma. The findings highlight the inconsistent nature of the current evidence base, partly due to the heterogeneity in response assessment and classification of non‐responders. To enable more standardized and accurate assessment of predictors and outcomes, patient‐centric definitions of response, such as CONFiRM [66], should be implemented in future severe asthma studies. Additionally, the limited data on differential predictors underscores the need for more head‐to‐head trials and *post hoc* analyses to identify and validate robust biologic response predictors. National reimbursement practices significantly influence treatment decisions; the limitations and accessibility of biologics in individual countries can affect both treatment initiation and switching choices. The present review is a step towards guiding healthcare providers in prescribing the most efficacious and thereby cost‐effective biologics for patients with severe asthma. This should allow more judicious resource allocation and treatment decision‐making, thereby benefiting both patients and healthcare systems.

## 5 Conclusion

This systematic review synthesised and evaluated the quality of evidence on predictors of response to anti‐IL5/5Rα, 4Rα, and anti‐TSLP for severe asthma. We found sufficient ‘moderate’ to ‘high’ quality evidence that: raised BEC levels (≥ 300 cells/μL) predict response to mepolizumab, dupilumab, and tezepelumab; elevated FeNO (> 40 ppb) predicts benralizumab response whilst FeNO 25–50 ppb is associated with better outcomes following dupilumab; OCS naïveté or low OCS dose (< 10 mg/day) predicts mepolizumab response, with low OCS dose (< 10 mg/day) predicting response to dupilumab; and better asthma control at baseline and post 3 months predicts mepolizumab and benralizumab response, respectively. There was some evidence, although inconsistent, suggesting that biologic naïveté and childhood‐onset asthma predict benralizumab response, while adult‐onset disease and higher BMI (> 30 kg/m^2^) predict positive and negative response to mepolizumab, respectively. Evidence for the other variables investigated was limited and predominantly ‘low’ quality owing to heterogeneous response definitions and imprecise effect estimates. This highlights the urgent need for larger, more standardised studies with homogenous response criteria to identify predictors beyond traditional markers, which can facilitate the selection of the optimal biologic for each patient with severe asthma.

## Author Contributions

Study concept and design: G.R., A.R. Literature searches, data extraction, and risk of bias assessment: A.R., P.D., D.C. Data analysis and synthesis: A.R. Drafting of the original manuscript: A.R., G.R. All authors provided critical review of the manuscript and approved the final version.

## Conflicts of Interest

Anna Rattu declares funding from the European Commission's Innovative Medicines Initiative 2 Joint Undertaking (JU) under grant agreement No. 831434 (3TR) and National Institute for Health and Care Research (NIHR) Southampton Biomedical Research Centre (BRC). Chris Brightling declares grants and consulting fees from 4D Pharma, Areteia, AstraZeneca (AZ), Chiesi, Genentech, GlaxoSmithKline (GSK), Mologic, Novartis, Regeneron Pharmaceuticals, Roche, and Sanofi paid to his institution; and support from the 3TR EU IMI2 project and NIHR BRC. Kian Fan Chung has received grants from MRC, EPSRC, and GSK paid to his institution; honoraria for speaking engagements for GSK, Novartis, AZ; remuneration for participation in Advisory Board meetings of GSK, AZ, Novartis, Roche, Merck, Trevi, Rickett‐Beckinson, Nocion, Shionogi, and participation in the Scientific Advisory Board of the Clean Breathing Institute supported by Haleon. Apostolos Bossios has received honoraria for lectures from AZ, GSK, Chiesi, and an institutional grant from AZ outside of the submitted work; is head of Assembly five for the European Respiratory Society (ERS), co‐chair of the Nordic Severe Asthma Network (NSAN), member of the steering committee of SHARP, ERS severe asthma Clinical Research Collaboration, and member of the Swedish National Airway Register steering committee. Arnaud Bourdin declares grants from Boehringer Ingelheim, AZ, and GSK; consulting fees from Boehringer Ingelheim, AZ, GSK, Novartis, Chiesi, Sanofi, Celltrion; honoraria from Sanofi, Regeneron, AZ, GSK, Novartis, Boehringer Ingelheim; support for attending meetings from AZ and Sanofi; participates in the advisory board for A.B. science. Ratko Djukanovic declares consulting fees for Synairgen plc, GSK, ZenasBio, Celltrion, ALK Abello; was past co‐chair of the ERS Clinical collaboration on severe asthma (SHARP); shares in Synairgen plc outside of the submitted work. Sven‐Erik Dahlén declares grants from AZ and GSK; consulting fees from Affibody, AZ, Cayman Chemical, GSK, Regeneron, and Sanofi; honoraria for lectures from AZ and Sanofi. Louise J Fleming has received a NIHR HTA grant outside of the submitted work; consulting fees from Sanofi, Regeneron, AZ, GSK; honoraria from AZ and Sanofi. All payments were made to the institution. Rekha Chaudhuri has received a grant from AZ for an investigator‐led study; lecture fees from GSK, AZ, TEVA, Chiesi, Sanofi; support for attending conferences from Chiesi, Sanofi, GSK; participation in Advisory Board Meetings for GSK, AZ, and Celltrion. Erik Melén declares advisory board fees from ALK and AZ outside the submitted work; lecture fees from ALK, AZ, Chiesi, and Sanofi outside the submitted study. Antoine Deschildre has received consulting fees from ALK, Stallergenes‐Greer, GSK, Sanofi, Regeneron, Aimmune Therapeutics, Celltrion, and Viatris; honoraria from Novartis, ALK, GSK, Sanofi, Aimmune Therapeutics, DBV Technologies, Viatris, and Stallergenes‐Greer; support for attending international congresses from Celltrion, ALK, Sanofi, Stallergenes‐Greer, Novartis, AZ, and Aimmune Therapeutics; participation on the Data Safety Monitoring Board for the BOOM study (ClinicalTrials.gov ID: NCT04045301). Charles Pilette has received grants from AZ, Chiesi, GSK, Sanofi; consulting fees from AstraZeneca, Chiesi, GSK; honoraria from ALK‐Abello, AstraZeneca, Chiesi, GSK, and Sanofi. All payments were made to his institution. Gerard H. Koppelman reports receiving research grants from the Netherlands Lung Foundation, Ubbo Emmius Foundation, Zon MW, H2020, and Vertex paid to his institution; consulting fees from AZ paid to his institution; institutional honoraria from AZ, Sanofi, and Boehringer Ingelheim; and is the chair and founder of the ExquAIro foundation. Andrew Exley declares being a minority shareholder in GSK. Ekaterina Khaleva has received grants from the Allergy and Inflammation Research (AAIR) charity and NIHR paid to her institution. Graham Roberts declares 3TR EU IMI2 funding and AZ fees paid to his institution. No other author has any conflicts of interest to declare.

## Supporting information


**Table S1:** Factors and consequence for grading the certainty of evidence.
**Table S2:** Inflammatory biomarkers as predictors of response to biological therapies for severe asthma.
**Table S3:** Lung function parameters as predictors of response to biological therapies for severe asthma.
**Table S4:** Clinical parameters as predictors of response to biological therapies for severe asthma.
**Table S5:** Co‐morbidities as predictors of response to biological therapies for severe asthma.
**Table S6:** Socio‐demographic characteristics as predictors of response to biological therapies for severe asthma.
**Table S7:** Preferred Reporting Items for Systematic Reviews and Meta‐Analyses (PRISMA) checklist.

## Data Availability

The data that supports the findings of this study are available in the Supporting Information—S1 of this article.

## References

[1] G. G. Brusselle and G. H. Koppelman , “Biologic Therapies for Severe Asthma,” New England Journal of Medicine 386, no. 2 (2022): 157–171. 35020986 10.1056/NEJMra2032506

[2] M. E. Hyland , B. Whalley , R. C. Jones , and M. Masoli , “A Qualitative Study of the Impact of Severe Asthma and Its Treatment Showing That Treatment Burden Is Neglected in Existing Asthma Assessment Scales,” Quality of Life Research 24, no. 3 (2015): 631–639. 25201169 10.1007/s11136-014-0801-x

[3] S. O'Neill , J. Sweeney , C. C. Patterson , et al., “The Cost of Treating Severe Refractory Asthma in the UK: An Economic Analysis From the British Thoracic Society Difficult Asthma Registry,” Thorax 70, no. 4 (2015): 376–378. 24917087 10.1136/thoraxjnl-2013-204114

[4] M. Kerkhof , T. N. Tran , J. B. Soriano , et al., “Healthcare Resource Use and Costs of Severe, Uncontrolled Eosinophilic Asthma in the UK General Population,” Thorax 73, no. 2 (2018): 116–124. 28918400 10.1136/thoraxjnl-2017-210531 PMC5801646

[5] A. Bourdin , L. Bjermer , C. Brightling , et al., “ERS/EAACI Statement on Severe Exacerbations in Asthma in Adults: Facts, Priorities and Key Research Questions,” European Respiratory Journal 54, no. 3 (2019): 1900900. 31467120 10.1183/13993003.00900-2019

[6] K. F. Chung , S. E. Wenzel , J. L. Brozek , et al., “International ERS/ATS Guidelines on Definition, Evaluation and Treatment of Severe Asthma,” European Respiratory Journal 43, no. 2 (2014): 343–373. 24337046 10.1183/09031936.00202013

[7] B. K. Chen , Y. T. Yang , and C. L. Bennett , “Why Biologics and Biosimilars Remain So Expensive: Despite Two Wins for Biosimilars, the Supreme Court's Recent Rulings Do Not Solve Fundamental Barriers to Competition,” Drugs 78, no. 17 (2018): 1777–1781. 30446980 10.1007/s40265-018-1009-0

[8] H. L. Gelhorn , Z. Balantac , C. S. Ambrose , Y. N. Chung , and B. Stone , “Patient and Physician Preferences for Attributes of Biologic Medications for Severe Asthma,” Patient Preference and Adherence 13 (2019): 1253–1268. 31440040 10.2147/PPA.S198953 PMC6667349

[9] E. S. Harvey , D. Langton , C. Katelaris , et al., “Mepolizumab Effectiveness and Identification of Super‐Responders in Severe Asthma,” European Respiratory Journal 55, no. 5 (2020): 1902420. 32139455 10.1183/13993003.02420-2019

[10] R. M. Niven , D. Saralaya , R. Chaudhuri , et al., “Impact of Omalizumab on Treatment of Severe Allergic Asthma in UK Clinical Practice: A UK Multicentre Observational Study (The APEX II Study),” BMJ Open 6, no. 8 (2016): e011857. 10.1136/bmjopen-2016-011857 PMC4985870 27507234

[11] K. Eger , J. A. Kroes , A. Ten Brinke , and E. H. Bel , “Long‐Term Therapy Response to Anti‐IL‐5 Biologics in Severe Asthma‐A Real‐Life Evaluation,” Journal of Allergy and Clinical Immunology. In Practice 9, no. 3 (2021): 1194–1200. 33069885 10.1016/j.jaip.2020.10.010

[12] M. Mukherjee , D. F. Forero , S. Tran , et al., “Suboptimal Treatment Response to Anti‐IL‐5 Monoclonal Antibodies in Severe Eosinophilic Asthmatics With Airway Autoimmune Phenomena,” European Respiratory Journal 56, no. 4 (2020): 2000117. 32444405 10.1183/13993003.00117-2020

[13] B. Bender , J. Oppenheimer , M. George , et al., “Assessment of Real‐World Escalation to Biologics in US Patients With Asthma,” Journal of Allergy and Clinical Immunology. In Practice 10, no. 11 (2022): 2941–2948. 35931363 10.1016/j.jaip.2022.07.016

[14] H. Kim , A. K. Ellis , D. Fischer , et al., “Asthma Biomarkers in the Age of Biologics,” Allergy, Asthma & Clinical Immunology 13, no. 1 (2017): 48. 10.1186/s13223-017-0219-4 PMC5691861 29176991

[15] F. Holguin , J. C. Cardet , K. F. Chung , et al., “Management of Severe Asthma: A European Respiratory Society/American Thoracic Society Guideline,” European Respiratory Journal 55, no. 1 (2020): 1900588. 31558662 10.1183/13993003.00588-2019

[16] Asthma GIf. Global Strategy for Asthma Management and Prevention, 2023.

[17] P. M. Hansbro , G. E. Kaiko , and P. S. Foster , “Cytokine/Anti‐Cytokine Therapy—Novel Treatments for Asthma?,” British Journal of Pharmacology 163, no. 1 (2011): 81–95. 21232048 10.1111/j.1476-5381.2011.01219.x PMC3085870

[18] R. Pettipher , M. G. Hunter , C. M. Perkins , et al., “Heightened Response of Eosinophilic Asthmatic Patients to the CRTH2 Antagonist OC000459,” Allergy 69, no. 9 (2014): 1223–1232. 24866478 10.1111/all.12451

[19] J. M. Harris , D. A. Wong , and A. V. Kapp , “Development of the Asthma Control Composite Outcome Measure to Predict Omalizumab Response,” Annals of Allergy, Asthma & Immunology 107, no. 3 (2011): 273. 10.1016/j.anai.2011.06.005 21875548

[20] E. Khaleva , A. Rattu , C. Brightling , et al., “Definitions of Non‐Response and Response to Biological Therapy for Severe Asthma: A Systematic Review,” ERJ Open Research 9, no. 3 (2023): 444–2022. 10.1183/23120541.00444-2022 PMC10152254 37143849

[21] J. W. Upham , C. le Lievre , D. J. Jackson , et al., “Defining a Severe Asthma Super‐Responder: Findings From a Delphi Process,” Journal of Allergy and Clinical Immunology. In Practice 9, no. 11 (2021): 3997–4004. 34271216 10.1016/j.jaip.2021.06.041

[22] A. I. Papaioannou , E. Fouka , K. Bartziokas , et al., “Defining Response to Therapy With Biologics in Severe Asthma: From Global Evaluation to Super Response and Remission,” Expert Review of Respiratory Medicine 17, no. 6 (2023): 481–493. 37318035 10.1080/17476348.2023.2226392

[23] Y. Li , X. Li , B. Zhang , et al., “Predictive Biomarkers for Response to Omalizumab in Patients With Severe Allergic Asthma: A Meta‐Analysis,” Expert Review of Respiratory Medicine 16 (2022): 1–11. 35730466 10.1080/17476348.2022.2092100

[24] D. Moher , A. Liberati , J. Tetzlaff , D. G. Altman , and The PRISMA Group , “Preferred Reporting Items for Systematic Reviews and Meta‐Analyses: The PRISMA Statement,” PLoS Medicine 6, no. 7 (2009): e1000097. 19621072 10.1371/journal.pmed.1000097 PMC2707599

[25] A. Rattu , E. Khaleva , C. Brightling , et al., “Identifying and Appraising Outcome Measures for Severe Asthma: A Systematic Review,” European Respiratory Journal 61, no. 4 (2023): 2201231. 36549712 10.1183/13993003.01231-2022

[26] E. Khaleva , A. Rattu , C. Brightling , et al., “Development of Core Outcome Measures Sets for Paediatric and Adult Severe Asthma (COMSA),” European Respiratory Journal 61, no. 4 (2023): 2200606. 36229046 10.1183/13993003.00606-2022 PMC10069873

[27] M. Ouzzani , H. Hammady , Z. Fedorowicz , and A. Elmagarmid , “Rayyan‐A Web and Mobile App for Systematic Reviews,” Systematic Reviews 5, no. 1 (2016): 210. 27919275 10.1186/s13643-016-0384-4 PMC5139140

[28] J. A. C. Sterne , J. Savović , M. J. Page , et al., “RoB 2: A Revised Tool for Assessing Risk of Bias in Randomised Trials,” BMJ (Clinical Research ed.) 366 (2019): l4898. 10.1136/bmj.l4898 31462531

[29] C. B. Terwee , C. A. C. Prinsen , A. Chiarotto , et al., “COSMIN Methodology for Evaluating the Content Validity of Patient‐Reported Outcome Measures: A Delphi Study,” Quality of Life Research 27, no. 5 (2018): 1159–1170. 29550964 10.1007/s11136-018-1829-0 PMC5891557

[30] L. B. Mokkink , H. C. W. de Vet , C. A. C. Prinsen , et al., “COSMIN Risk of Bias Checklist for Systematic Reviews of Patient‐Reported Outcome Measures,” Quality of Life Research 27, no. 5 (2018): 1171–1179. 29260445 10.1007/s11136-017-1765-4 PMC5891552

[31] G. Guyatt , A. D. Oxman , E. A. Akl , et al., “GRADE Guidelines: 1. Introduction‐GRADE Evidence Profiles and Summary of Findings Tables,” Journal of Clinical Epidemiology 64, no. 4 (2011): 383–394. 21195583 10.1016/j.jclinepi.2010.04.026

[32] L. Bergantini , M. D'Alessandro , P. Cameli , et al., “Personalized Approach of Severe Eosinophilic Asthma Patients Treated With Mepolizumab and Benralizumab,” International Archives of Allergy and Immunology 181, no. 10 (2020): 746–753. 32731216 10.1159/000508936

[33] M. B. Bilò , M. Martini , L. Antonicelli , et al., “Severe Asthma: Follow‐Up After One Year From the Italian Registry on Severe Asthma (IRSA),” European Annals of Allergy and Clinical Immunology 55, no. 5 (2023): 199–211. 37462932 10.23822/EurAnnACI.1764-1489.304

[34] M. Caminati , A. Marcon , G. Guarnieri , et al., “Benralizumab Efficacy in Late Non‐Responders to Mepolizumab and Variables Associated With Occurrence of Switching: A Real‐Word Perspective,” Journal of Clinical Medicine 12, no. 5 (2023): 1836. 36902623 10.3390/jcm12051836 PMC10002580

[35] G. L. Chupp , E. S. Bradford , F. C. Albers , et al., “Efficacy of Mepolizumab Add‐On Therapy on Health‐Related Quality of Life and Markers of Asthma Control in Severe Eosinophilic Asthma (MUSCA): A Randomised, Double‐Blind, Placebo‐Controlled, Parallel‐Group, Multicentre, Phase 3b Trial,” Lancet Respiratory Medicine 5, no. 5 (2017): 390–400. 28395936 10.1016/S2213-2600(17)30125-X

[36] C. Crimi , R. Campisi , G. Cacopardo , et al., “Real‐Life Effectiveness of Mepolizumab in Patients With Severe Refractory Eosinophilic Asthma and Multiple Comorbidities,” World Allergy Organization Journal 13, no. 9 (2020): 100462. 32994855 10.1016/j.waojou.2020.100462 PMC7508691

[37] W. C. G. Fong , A. Azim , D. Knight , et al., “Real‐World Omalizumab and Mepolizumab Treated Difficult Asthma Phenotypes and Their Clinical Outcomes,” Clinical and Experimental Allergy 51, no. 8 (2021): 1019–1032. 33866615 10.1111/cea.13882

[38] J. E. Kavanagh , G. d'Ancona , M. Elstad , et al., “Real‐World Effectiveness and the Characteristics of a “Super‐Responder” to Mepolizumab in Severe Eosinophilic Asthma,” Chest 158, no. 2 (2020): 491–500. 32275980 10.1016/j.chest.2020.03.042

[39] C. Pilette , G. W. Canonica , R. Chaudhuri , et al., “REALITI‐A Study: Real‐World Oral Corticosteroid‐Sparing Effect of Mepolizumab in Severe Asthma,” Journal of Allergy and Clinical Immunology: In Practice 10 (2022): 2646–2656. 35753668 10.1016/j.jaip.2022.05.042

[40] C. Reilly , A. Raja , P. Anilkumar , et al., “The Clinical Effectiveness of Mepolizumab Treatment in Severe Eosinophilic Asthma; Outcomes From Four Years Cohort Evaluation,” Journal of Asthma 61, no. 6 (2024): 561–573. 10.1080/02770903.2023.2294908 38088937

[41] H. Sasano , N. Harada , S. Harada , et al., “Pretreatment Circulating MAIT Cells, Neutrophils, and Periostin Predicted the Real‐World Response After 1‐Year Mepolizumab Treatment in Asthmatics,” Allergology International 73, no. 1 (2024): 94–106. 37336695 10.1016/j.alit.2023.06.001

[42] D. Thomas , E. S. Harvey , V. M. McDonald , et al., “Mepolizumab and Oral Corticosteroid Stewardship: Data From the Australian Mepolizumab Registry,” Journal of Allergy and Clinical Immunology: In Practice 9, no. 7 (2021): 2715–2724. 33545399 10.1016/j.jaip.2021.01.028

[43] M. Al‐Ahmad , A. Ali , and A. Maher , “Factors Influencing Poor Response to Type 2 Targeted Therapies in Severe Asthma: A Retrospective Cohort Study,” BMC Pulmonary Medicine 23, no. 1 (2023): 490. 38053108 10.1186/s12890-023-02786-w PMC10699072

[44] J. E. Kavanagh , A. P. Hearn , J. Dhariwal , et al., “Real‐World Effectiveness of Benralizumab in Severe Eosinophilic Asthma,” Chest 159, no. 2 (2021): 496–506. 32882249 10.1016/j.chest.2020.08.2083

[45] J. A. Kroes , K. de Jong , S. Hashimoto , et al., “Clinical Response to Benralizumab Can Be Predicted by Combining Clinical Outcomes at 3 Months With Baseline Characteristics,” ERJ Open Research 9, no. 2 (2023): 559–2022. 10.1183/23120541.00559-2022 PMC10086738 37057095

[46] Y. Sandhu , N. Harada , H. Sasano , et al., “Pretreatment Frequency of Circulating Th17 Cells and FeNO Levels Predicted the Real‐World Response After 1 Year of Benralizumab Treatment in Patients With Severe Asthma,” Biomolecules 13, no. 3 (2023): 538. 36979473 10.3390/biom13030538 PMC10046637

[47] H. Watanabe , T. Shirai , K. Hirai , et al., “Blood Eosinophil Count and FeNO to Predict Benralizumab Effectiveness in Real‐Life Severe Asthma Patients,” Journal of Asthma 59 (2021): 1796–1804. 10.1080/02770903.2021.1963769 34348060

[48] C. Domingo , J. F. Maspero , M. Castro , et al., “Dupilumab Efficacy in Steroid‐Dependent Severe Asthma by Baseline Oral Corticosteroid Dose,” Journal of Allergy and Clinical Immunology. In Practice 10, no. 7 (2022): 1835–1843. 35398549 10.1016/j.jaip.2022.03.020

[49] K. F. Rabe , P. Nair , G. Brusselle , et al., “Efficacy and Safety of Dupilumab in Glucocorticoid‐Dependent Severe Asthma,” New England Journal of Medicine 378, no. 26 (2018): 2475–2485. 29782224 10.1056/NEJMoa1804093

[50] K. F. Rabe , P. Nair , J. F. Maspero , et al., “The Effect of Dupilumab on Lung Function Parameters in Patients With Oral Corticosteroid‐Dependent Severe Asthma,” Respiratory Medicine: X 2 (2020): 100010.

[51] M. E. Wechsler , A. Menzies‐Gow , C. E. Brightling , et al., “Evaluation of the Oral Corticosteroid‐Sparing Effect of Tezepelumab in Adults With Oral Corticosteroid‐Dependent Asthma (SOURCE): A Randomised, Placebo‐Controlled, Phase 3 Study,” Lancet Respiratory Medicine 10 (2022): 650–660. 35364018 10.1016/S2213-2600(21)00537-3

[52] H. G. Ortega , M. C. Liu , I. D. Pavord , et al., “Mepolizumab Treatment in Patients With Severe Eosinophilic Asthma,” New England Journal of Medicine 371, no. 13 (2014): 1198–1207. 25199059 10.1056/NEJMoa1403290

[53] J. Corren , J. R. Parnes , L. W. Wang , et al., “Tezepelumab in Adults With Uncontrolled Asthma,” New England Journal of Medicine 377, no. 10 (2017): 936–946. 28877011 10.1056/NEJMoa1704064

[54] E. R. Bleecker , J. M. FitzGerald , P. Chanez , et al., “Efficacy and Safety of Benralizumab for Patients With Severe Asthma Uncontrolled With High‐Dosage Inhaled Corticosteroids and Long‐Acting β2‐Agonists (SIROCCO): A Randomised, Multicentre, Placebo‐Controlled Phase 3 Trial,” Lancet 388, no. 10056 (2016): 2115–2127. 27609408 10.1016/S0140-6736(16)31324-1

[55] J. M. FitzGerald , E. R. Bleecker , P. Nair , et al., “Benralizumab, an Anti‐Interleukin‐5 Receptor α Monoclonal Antibody, as Add‐On Treatment for Patients With Severe, Uncontrolled, Eosinophilic Asthma (CALIMA): A Randomised, Double‐Blind, Placebo‐Controlled Phase 3 Trial,” Lancet 388, no. 10056 (2016): 2128–2141. 27609406 10.1016/S0140-6736(16)31322-8

[56] M. Castro , J. Corren , I. D. Pavord , et al., “Dupilumab Efficacy and Safety in Moderate‐To‐Severe Uncontrolled Asthma,” New England Journal of Medicine 378, no. 26 (2018): 2486–2496. 29782217 10.1056/NEJMoa1804092

[57] A. G. Fiocchi , W. Phipatanakul , R. S. Zeiger , et al., “Dupilumab Leads to Better‐Controlled Asthma and Quality of Life in Children: The VOYAGE Study,” European Respiratory Journal 62, no. 5 (2023): 2300558. 37734856 10.1183/13993003.00558-2023 PMC10620476

[58] A. Menzies‐Gow , G. Colice , J. M. Griffiths , et al., “NAVIGATOR: A Phase 3 Multicentre, Randomized, Double‐Blind, Placebo‐Controlled, Parallel‐Group Trial to Evaluate the Efficacy and Safety of Tezepelumab in Adults and Adolescents With Severe, Uncontrolled Asthma,” Respiratory Research 21, no. 1 (2020): 266. 33050934 10.1186/s12931-020-01526-6 PMC7550847

[59] H. G. Ortega , S. W. Yancey , B. Mayer , et al., “Severe Eosinophilic Asthma Treated With Mepolizumab Stratified by Baseline Eosinophil Thresholds: A Secondary Analysis of the DREAM and MENSA Studies,” Lancet Respiratory Medicine 4, no. 7 (2016): 549–556. 27177493 10.1016/S2213-2600(16)30031-5

[60] J. M. FitzGerald , E. R. Bleecker , A. Menzies‐Gow , et al., “Predictors of Enhanced Response With Benralizumab for Patients With Severe Asthma: Pooled Analysis of the SIROCCO and CALIMA Studies,” Lancet Respiratory Medicine 6, no. 1 (2018): 51–64. 28919200 10.1016/S2213-2600(17)30344-2

[61] R. Buhl , E. Bel , A. Bourdin , et al., “Effective Management of Severe Asthma With Biologic Medications in Adult Patients: A Literature Review and International Expert Opinion,” Journal of Allergy and Clinical Immunology. In Practice 10, no. 2 (2022): 422–432. 34763123 10.1016/j.jaip.2021.10.059

[62] E. Heffler , G. Paoletti , V. Giorgis , et al., “Real‐Life Studies of Biologics Used in Asthma Patients: Key Differences and Similarities to Trials,” Expert Review of Clinical Immunology 15, no. 9 (2019): 951–958. 31389304 10.1080/1744666X.2019.1653758

[63] C. Pelaia , E. Heffler , C. Crimi , et al., “Interleukins 4 and 13 in Asthma: Key Pathophysiologic Cytokines and Druggable Molecular Targets,” Frontiers in Pharmacology 13 (2022): 851940. 35350765 10.3389/fphar.2022.851940 PMC8957960

[64] C. Moermans , C. Brion , G. Bock , et al., “Sputum Type 2 Markers Could Predict Remission in Severe Asthma Treated With Anti‐IL‐5,” Chest 163, no. 6 (2023): 1368–1379. 36740095 10.1016/j.chest.2023.01.037

[65] P. Howarth , G. Chupp , L. M. Nelsen , et al., “Severe Eosinophilic Asthma With Nasal Polyposis: A Phenotype for Improved Sinonasal and Asthma Outcomes With Mepolizumab Therapy,” Journal of Allergy and Clinical Immunology 145, no. 6 (2020): 1713–1715. 32084443 10.1016/j.jaci.2020.02.002

[66] E. B. C. Khaleva and T. Eiwegger , “Patient‐Centred Composite Index for Response in Asthma (CONFiRM) to Biological Therapy for Children and Adults,” ERJ 65, no. 3 (2024): 2400691.

[67] D. M. Kent , E. Steyerberg , and D. van Klaveren , “Personalized Evidence Based Medicine: Predictive Approaches to Heterogeneous Treatment Effects,” BMJ (Clinical Research ed.) 363 (2018): k4245. 10.1136/bmj.k4245 PMC6889830 30530757

[68] C. Porsbjerg , A. H. Maitland‐van der Zee , G. Brusselle , et al., “3TR: A Pan‐European Cross‐Disease Research Consortium Aimed at Improving Personalised Biological Treatment of Asthma and COPD,” European Respiratory Journal 58, no. 4 (2021): 2102168. 34675035 10.1183/13993003.02168-2021

[69] C. L. Gaberino , L. B. Bacharier , and D. J. Jackson , “Controversies in Allergy: Are Biologic Treatment Responses in Severe Asthma the Same in Adults and Children?,” Journal of Allergy and Clinical Immunology. In Practice 11, no. 9 (2023): 2673–2682. 37517797 10.1016/j.jaip.2023.07.028

[70] T. S. C. Hinks , S. J. Levine , and G. G. Brusselle , “Treatment Options in Type‐2 Low Asthma,” European Respiratory Journal 57, no. 1 (2021): 2000528. 32586877 10.1183/13993003.00528-2020 PMC7116624

[71] J. Charriot , E. Ahmed , and A. Bourdin , “Local Targeting of TSLP: Feat or Defeat,” European Respiratory Journal 61, no. 3 (2023): 2202389. 36894191 10.1183/13993003.02389-2022

[72] G. M. Gauvreau , J. M. Hohlfeld , J. M. FitzGerald , et al., “Inhaled Anti‐TSLP Antibody Fragment, Ecleralimab, Blocks Responses to Allergen in Mild Asthma,” European Respiratory Journal 61, no. 3 (2023): 2201193. 36822634 10.1183/13993003.01193-2022 PMC9996823

