# Supplementary material for: Predictors of Response to Biologics for Severe Asthma: A Systematic Review and Meta‐Analysis
Source: Allergy. 2025 Sep 16;81(1):24–55. doi: 10.1111/all.70031 (PMC12773690; doi:10.1111/all.70031)
Supplement: Supplementary file 1 — Table S1: Factors and consequence for grading the certainty of evidence. Table S2: Inflammatory biomarkers as predictors of response to biological therapies for severe asthma. Table S3: Lung function parameters as predictors of response to biological therapies for severe asthma. Table S4: Clinical parameters as predictors of response to biological therapies for severe asthma. Table S5: Co‐morbidities as predictors of response to biological therapies for severe asthma. Table S6: Socio‐demographic characteristics as predictors of response to biological therapies for severe asthma. Table S7: Preferred Reporting Items for Systematic Reviews and Meta‐Analyses (PRISMA) checklist. [file ALL-81-24-s001.docx]

**ONLINE SUPPLEMENT**

**Predictors of response to biologics for severe asthma: a systematic review and meta-analysis**

**Authors:** Anna Rattu, Piers Dixey, David Charles, Chris Brightling, Kian Fan Chung, Apostolos Bossios, Arnaud Bourdin, Ratko Djukanovic, Sven-Erik Dahlén, Louise Fleming, Rekha Chaudhuri, Erik Melén, Antoine Deschildre, Charles Pilette, Gerard H. Koppelman, Andrew Exley, Freja Anckers, Sarah Miller, Hanna Nielsen, Clare Williams, Ekaterina Khaleva, Graham Roberts on behalf of the 3TR consortium Respiratory Work Package.

**Affiliations:**

Anna Rattu: 1. Department of Human Development and Health, Faculty of Medicine, School of Health Sciences, Institute of Environmental and Life Sciences, University of Southampton, UK; 2. NIHR Southampton Biomedical Research Centre, University Hospital Southampton NHS Foundation Trust, Southampton, UK. ORCID: 0000-0002-7497-9552.

Piers H A Dixey: National Heart Lung institute, Imperial College, London, UK; Royal Brompton Hospital,

London, UK. ORCID: 0000-0003-3080-8978.

David Charles: Academic Clinical Medicine, Southampton General Hospital, Southampton, UK.

Chris Brightling: Institute for Lung Health, Leicester NIHR BRC, University of Leicester, UK. ORCID: 0000-0002-9345-4903.

Kian Fan Chung: National Heart & Lung Institute, Imperial College London, London. UK. ORCID: 0000-0001-7101-1426.

Apostolos Bossios: 1) Karolinska Severe Asthma Center, Department of Respiratory Medicine and Allergy, Karolinska University Hospital, Stockholm, Sweden 2) Division of Lung and Airway Research, Institute of Environmental Medicine, Karolinska Institute, Stockholm, Sweden 3) Lung laboratory, Center for Molecular Medicine, Karolinska University Hospital, Stockholm, Sweden.

Arnaud Bourdin: PhyMedExp, University of Montpellier, Montpellier, France. ORCID 0000-0002-4645-5209.

Rekha Chaudhuri: School of Infection & Immunity, University of Glasgow, Glasgow, UK.

Sven-Erik Dahlén: Department of Respiratory Medicine and Allergy, Karolinska University Hospital, Huddinge, Department of Medicine, Huddinge, Karolinska Institutet, and Institute of Environmental Medicine, Karolinska Institutet, Stockholm, Sweden.

Ratko Djukanovic: NIHR Southampton Biomedical Research Centre, University Hospital Southampton,

Clinical and Experimental Sciences, Faculty of Medicine, University of Southampton, Sir Henry Wellcome Laboratories, Southampton, UK. ORCID: 0000-0001-6039-5612.

Antoine Deschildre: CHU Lille, Unité de Pneumologie et Allergologie Pédiatrique, Hôpital Jeanne de

Flandre F-59000 Lille, France; Univ. Lille, U1019 - UMR 8204 - CIIL - Center for Infection and Immunity of Lille, Lille, France.

Louise Fleming: Imperial College Healthcare Trust and National Heart and Lung Institute, Imperial College, London. ORCID: 0000-0002-7268-7433.

Gerard H. Koppelman: University of Groningen, University Medical Center Groningen, Beatrix Children’s Hospital, Department of Pediatric Pulmonology and Pediatric Allergology, Groningen, the Netherlands; University of Groningen, University Medical Center Groningen, Groningen Research Institute for Asthma and COPD (GRIAC), Groningen, the Netherlands. ORCID 0000-0001-8567-3252.

Erik Melén: Department of Clinical Science and Education Södersjukhuset, Karolinska Institutet,

Stockholm, Sweden. ORCID: 0000-0002-8248-0663.

Charles Pilette: Department of Pulmonology, Cliniques universitaires Saint-Luc & pole of lung, nose and skin research (LUNS), Institute of experimental and clinical research (IREC), UCLouvain, Brussels, Belgium.

Freja Anckers: Patient and Public Involvement Representative, Sweden.

Sarah Miller: Patient and Public Involvement Representative, UK.

Hanna Nielsen: Patient and Public Involvement Representative, Sweden. Falu Lasarett, Region Dalarna, Sweden.

Clare Williams: European Lung Foundation, Sheffield, UK. ORCID 0000-0001-9446-0339.

Andrew Exley: Adept Biologica Consulting Limited, London, UK. ORCID: 0000-0002-2628-6129.

Ekaterina Khaleva: 1. Human Development and Health, Faculty of Medicine, University of Southampton, Southampton, UK; 2. Royal Hampshire County Hospital, Winchester, UK. ORCID: 0000-0002-2220-7745.

Graham Roberts: Clinical and Experimental Sciences and Human Development in Health, Faculty of

Medicine, University of Southampton, Southampton, UK; Paediatric Allergy and Respiratory Medicine,

University Hospital Southampton NHS Foundation Trust, Southampton, UK. ORCID: 0000-0003-2252-1248.

**Correspondence address:** Professor Graham Roberts, Paediatric Allergy and Respiratory Medicine, University Child Health (MP803), University Hospital, Southampton NHS Foundation Trust, Tremona Road, Southampton SO16 6YD, UK. Tel.: +44 (0) 2380796160, E-mail: [g.c.roberts@soton.ac.uk](mailto:g.c.roberts@soton.ac.uk).

Table of Contents

[Table of Contents 3](#_Toc187174055)

[I. Quality appraisal strategy 4](#_Toc187174056)

[II. Search strategy 5](#_Toc187174061)

[Database: Embase (OVID) 5](#_Toc187174062)

[Database: Medline (OVID) 7](#_Toc187174063)

[Database: Cinahl (EBSCOHOST) 8](#_Toc187174064)

[Database: Web of Science 9](#_Toc187174065)

[CENTRAL (via the Cochrane library) – CT.GOV and ICTRP registries 11](#_Toc187174066)

[III. Narrative summary of evidence for predictors of response to biologics 13](#_Toc187174067)

[IV. PRISMA checklist 36](#_Toc187174068)

[Reference list 39](#_Toc187174069)

# Quality appraisal strategy

**Table S1.** Factors and consequence for grading the certainty of evidence.

| Factor | Consequence |
| --- | --- |
| **Factors that can reduce the quality of evidence*** | |
| 1. Limitations in study design or execution (Risk of bias) | **↓**1 level: serious risk of bias e.g. flawed measurement or definition of:   - Predictor: heterogeneity in measurement or definition across studies (e.g. different thresholds for ‘high’ blood eosinophil count (BEC)), or lack of reporting of comparison between thresholds for continuous variables (e.g. >300 vs <300 BEC) or groups for dichotomous variables (e.g. smokers vs non-smokers) - Outcome: heterogeneity in response definitions across studies, or use of surrogate endpoints in definition of clinical response.   **↓** 2 levels: very serious risk of bias e.g.:   - Flawed measurement or definition of predictor and outcome - Lack of quantitative outcome data or only p-values reported. |
| 1. Inconsistency of results | **↓**1 level: contrasting results between two studies (e.g. one shows that a variable is predictive of (non-) response whereas other does not).  **↓** 2 levels: ≥ three studies with contrasting results. |
| 1. Imprecision | **↓**1 level: small study (n<100)**^†^** with wide CI, or study reporting other indicators of poor predictive ability e.g. AUC 0.6-0.7, sensitivity 70-79%, or specificity 70-79%.  **↓** 2 levels: CI includes 1 (no effect), or other indicators of very poor predictive ability e.g. AUC <0.6, sensitivity <70%, or specificity <70%. |
| Publication bias | NA (addressed by imprecision) |
| **Factors that can increase the quality of evidence** | |
| 1. Large magnitude of effect | **↑** 1 level: one study with clinically meaningful change in response outcome**^††^**.  **↑** 2 levels: ≥ two studies with clinically meaningful change in response outcome**^††^**. |
| All plausible confounding would reduce demonstrated effect or increase the effect if no effect was observed | NA |
| 1. Dose-response gradient | **↑** 1 level: one study demonstrating dose-response relationship between predictor level and response outcome.  **↑** 2 levels: ≥ two studies demonstrating dose-response relationship between predictor level and response outcome. |

*: Indirectness of evidence not included as eligible studies only included participants with severe asthma. †: Cut off agreed arbitrarily by review team. ††: Clinically meaningful change in outcomes based on literature: decrease or increase by 0.5 exacerbations per year; ≥50% reduction or increase in maintenance oral corticosteroids (mOCS); Asthma Control Test (ACT) – for adults 3 points (MCID)^1^, for patients aged 12–18 y 2 points (MCID); Asthma Control Questionnaire-5,-6, and -7: for adults 0.5 points^2^ (MCID), for paediatric ACQ-6 (symptoms and rescue medication use) 0.6 points (MCID)^3^, for ACQ-5 (symptoms only) 0.7 points^3^; St George’s Respiratory Questionnaire (SGRQ): 4 points (MCID)^4^; FEV_1_ MID agreed by a Guideline Development group: 0.20 L^5^

# Search strategy

## Database: Embase (OVID)

**Population**

1. asthma/ or allergic asthma/ or aspirin exacerbated respiratory disease/ or asthmatic state/ or exercise induced asthma/ or experimental asthma/ or extrinsic asthma/ or intrinsic asthma/ or mild intermittent asthma/ or mild persistent asthma/ or moderate persistent asthma/ or nocturnal asthma/ or occupational asthma/ or severe persistent asthma/

2. asthma*.ti,ab.

3. 1 or 2

**Intervention**

4. omalizumab.mp. or exp omalizumab/ or Xolair.mp.

5. mepolizumab.mp. or exp mepolizumab/ or nucala.mp. or Bosatria.mp.

6. reslizumab.mp. or reslizumab/ or cinqair.mp. or cinqaero.mp. or Cinqaer.mp. or Cinquil.mp.

7. benralizumab.mp. or exp benralizumab/ or fasenra.mp.

8. dupilumab.mp. or exp dupilumab/ or Dupixent.mp.

9. tralokinumab.mp. or exp tralokinumab/

10. lebrikizumab.mp. or exp lebrikizumab/ or TNX-650.mp.

11. tezepelumab.mp. or exp tezepelumab/

12. brodalumab.mp. or exp brodalumab/ or siliq.mp. or kyntheum.mp.

13. ligelizumab.mp. or exp ligelizumab/ or QGE031.mp.

14. Pitrakinra.mp. or pitrakinra/ or Aerovant.mp.

15. 4 or 5 or 6 or 7 or 8 or 9 or 10 or 11 or 12 or 13 or 14

**Study inclusion filter**

16. Clinical trial/ or Randomized controlled trial/ or Randomization/ or Single blind procedure/ or Double blind procedure/ or Crossover procedure/ or Placebo/ or Prospective study/ or comparative study/

17. Randomi?ed controlled trial*.mp.

18. Rct.mp.

19. (clinic* adj2 trial*).mp.

20. ((allocat* adj2 random*) or (assign* adj2 random*)).mp.

21. ((singl* or doubl* or trebl* or tripl*) adj2 (blind* or mask*)).mp.

22. Placebo*.mp.

23. (prospective adj2 (study or studies)).mp.

24. (comparative adj2 (study or studies)).mp.

25. ((cross-over or crossover) adj2 (study or studies or design or procedure)).mp.

26. 16 or 17 or 18 or 19 or 20 or 21 or 22 or 23 or 24 or 25

27. exp controlled study/ or exp longitudinal study/ or exp retrospective study/ or exp cohort analysis/ or exp follow up/ or exp case control study/

28. (Cohort adj2 (study or studies or analysis)).mp.

29. (control* adj3 (study or studies or trial)).mp.

30. (follow up adj1 (study or studies)).mp.

31. (observational adj1 (study or studies)).mp.

32. (epidemiologic* adj1 (study or studies)).mp.

33. (Real-World or Realworld).mp.

34. (Real-Life or reallife).mp.

35. 27 or 28 or 29 or 30 or 31 or 32 or 33 or 34

36. 26 or 35

**Study exclusion filter – 1**

37. editorial/ or review/ or case report/ or case report*.mp.

38. editorial*.mp.

39. conference abstract*.mp.

40. conference paper*.mp. or conference paper/ or conference abstract/

41. ((systematic or narrative) adj2 review*).mp. or "systematic review"/

42. 37 or 38 or 39 or 40 or 41

**Combination**

43. 3 and 15 and 36

44. 43 not 42

**Study exclusion filter – 2**

45. (exp animal/ or nonhuman/) not exp human/

46. 44 not 45

**Language limit**

47. limit 46 to english language

**Publication year limit**

48. limit 47 to yr="1990 -Current"

## Database: Medline (OVID)

**Population**

1. exp Asthma, Aspirin-Induced/ or exp Asthma, Exercise-Induced/ or exp Asthma/ or exp Asthma, Occupational/ or asthma*.ti,ab.

**Intervention**

2. omalizumab.mp. or Omalizumab/ or Xolair.mp.

3. (mepolizumab or nucala or Bosatria).mp.

4. (reslizumab or cinqair or cinqaero or Cinqaer or Cinquil).mp.

5. (benralizumab or fasenra).mp.

6. (dupilumab or Dupixent).mp.

7. tralokinumab.mp.

8. (lebrikizumab or TNX-650).mp.

9. tezepelumab.mp.

10. (brodalumab or siliq or kyntheum).mp.

11. (ligelizumab or QGE031).mp.

12. (Pitrakinra or Aerovant).mp.

13. 2 or 3 or 4 or 5 or 6 or 7 or 8 or 9 or 10 or 11 or 12

**Study inclusion filter**

14. Clinical trial/ or exp Clinical Trials as Topic/ or Randomized controlled trials as Topic/ or Randomized controlled trial/ or Random allocation/ or Double blind method/ or Single blind method/ or cross-over studies/ or Placebos/ or Prospective Studies/ or Comparative Study/

15. Randomi?ed controlled trial*.mp.

16. Rct.mp.

17. (clinic* adj2 trial*).mp.

18. ((singl* or doubl* or trebl* or tripl*) adj2 (blind* or mask*)).mp.

19. ((allocat* adj2 random*) or (assign* adj2 random*)).mp.

20. Placebo*.mp.

21. (prospective adj2 (study or studies)).mp.

22. (comparative adj2 (study or studies)).mp.

23. ((cross-over or crossover) adj2 (study or studies or design or procedure)).mp.

24. 14 or 15 or 16 or 17 or 18 or 19 or 20 or 21 or 22 or 23

25. exp case-control studies/ or exp Longitudinal Studies/ or exp retrospective studies/ or exp Cohort Studies/ or exp Controlled Before-After Studies/ or exp Follow-Up Studies/

26. (Cohort adj2 (study or studies or analysis)).mp.

27. (control* adj3 (study or studies or trial)).mp.

28. (follow up adj1 (study or studies)).mp.

29. (observational adj1 (study or studies)).mp.

30. (epidemiologic* adj1 (study or studies)).mp.

31. (Real-World or Realworld).mp.

32. (Real-Life or reallife).mp.

33. 25 or 26 or 27 or 28 or 29 or 30 or 31 or 32

34. 24 or 33

**Study exclusion filter – 1**

35. editorial/ or review/ or case report/ or case report*.mp.

36. (editorial* or conference abstract* or conference paper*).mp.

37. ((systematic or narrative) adj2 review*).mp. or "systematic review"/

38. 35 or 36 or 37

**Combination**

39. 1 and 13 and 34

40. 39 not 38

**Study exclusion filter – 2**

41. 40 not (Animals/ not (Animals/ and Humans/))

**Language limit**

42. limit 41 to english language

**Publication year limit**

43. limit 42 to yr="1990 -Current"

## Database: Cinahl (EBSCOHOST)

**Population**

1. (MH "Asthma+") OR (MH "Asthma, Occupational") OR (MH "Asthma, Exercise-Induced") OR TI asthma* OR AB asthma*

**Intervention**

2. omalizumab OR xolair OR mepolizumab OR nucala OR bosatria OR reslizumab OR cinqair OR cinqaero OR cinqaer OR cinquil OR benralizumab OR fasenra OR dupilumab OR dupixent OR tralokinumab OR lebrikizumab OR "TNX-650" OR tezepelumab OR brodalumab OR siliq OR kyntheum OR ligelizumab OR QGE031 OR pitrakinra OR aerovant

**Study inclusion filter**

3. (MH "Clinical Trials") OR (MH "Randomized Controlled Trials") OR (MH "Random Assignment") OR (MH "Single-Blind Studies") OR (MH "Double-Blind Studies") OR (MH "Crossover Design") OR (MH "Placebos") OR (MH "Prospective Studies") OR (MH "Comparative Studies") OR "Randomi?ed controlled trial*" OR Rct OR (clinic* N2 trial*) OR ((allocat* N2 random*) OR (assign* N2 random*)) OR ((singl* OR doubl* OR trebl* OR tripl*) N2 (blind* OR mask*)) OR Placebo* OR ((prospective N2 (study OR studies)) OR (comparative N2 (study OR studies)) OR ((cross-over OR crossover) N2 (study OR studies OR design OR procedure))

4. (MH "Case Control Studies+") OR (MH "Retrospective Panel Studies") OR (MH "Controlled Before-After Studies") OR (cohort* N2 (study OR studies OR analysis)) OR (control* N3 (study OR studies OR trial)) OR (("follow up" OR observational OR epidemiologic*) N1 (study OR studies)) OR "Real-World" OR Realworld OR "Real-Life" OR reallife

**Study exclusion filter – 1**

5. (MH "Literature Review") OR (MH "Scoping Review") OR PT "Systematic Review" OR PT "review" OR PT "editorial" OR PT "proceedings"

**Combination**

6. S1 AND S2 AND S3 AND S4

7. S6 NOT S5

**Study exclusion filter – 2**

8. (MH "animals+") NOT (MH "human")

**Language limit**

9. S7 NOT S8 (Limiters - English Language)

**Publication year limit**

10. S7 NOT S8 (Limiters - Published date: 19900101-20220731)

## Database: Web of Science

**Population**

1. TS=(asthma*)

**Intervention**

2. TS=(omalizumab) OR TS=(xolair) OR TS=(mepolizumab) OR TS=(nucala) OR TS=(bosatria) OR TS=(reslizumab) OR TS=(cinqair) OR TS=(cinqaero) OR TS=(cinqaer) OR TS=(cinquil) OR TS=(benralizumab) OR TS=(fasenra) OR TS=(dupilumab) OR TS=(dupixent) OR TS=(tralokinumab) OR TS=(lebrikizumab) OR TS=(TNX-650) OR TS=(tezepelumab) OR TS=(brodalumab) OR TS=(siliq) OR TS=(kyntheum) OR TS=(ligelizumab) OR TS=(QGE031) OR TS=(Pitrakinra) OR TS=(aerovant)

**Study inclusion filter**

3. TS=(clinic* NEAR/2 trial*) OR TS=(randomi?ation) OR TS=((singl* OR doubl* OR trebl* OR tripl*) NEAR/2 (blind* OR mask*)) OR TS=(Placebo*) OR TS=("Randomi?ed Controlled Trial*") OR TS=(Rct) OR TS=((allocat* NEAR/2 random*) OR (assign* NEAR/2 random*)) OR TS=(prospective NEAR/2 (study OR studies)) OR TS=(comparative NEAR/2 (study OR studies)) OR TS=(("cross-over" OR crossover) NEAR/2 (study OR studies OR design OR procedure))

4. TS=(cohort* NEAR/2 (study OR studies OR analysis)) OR TS=(control* NEAR/3 (study OR studies OR trial)) OR TS=(("follow up" OR observational OR epidemiologic*) NEAR/1 (study OR studies)) OR TS=("Real-World") OR TS=(Realworld) OR TS=("Real-Life") OR TS=(reallife)

**Combination**

5. #1 AND #2 AND #3 AND #4

**Study exclusion filter – 1**

6. (#5 NOT TS=((animal*) NOT (human* OR patient*)))

**Study exclusion filter – 2**

7. (#5 NOT TS=((animal*) NOT (human* OR patient*))) AND

Refined by: [excluding] DOCUMENT TYPES: (MEETING ABSTRACTS OR EDITORIAL MATERIALS OR PROCEEDINGS PAPERS OR REVIEW ARTICLES)

**Language limit**

8. (#5 NOT TS=((animal*) NOT (human* OR patient*))) AND

Refined by: [excluding] DOCUMENT TYPES: (MEETING ABSTRACTS OR EDITORIAL MATERIALS OR PROCEEDINGS PAPERS OR REVIEW ARTICLES)

AND LANGUAGE: (English)

**Publication year limit**

8. (#5 NOT TS=((animal*) NOT (human* OR patient*))) AND

Refined by: [excluding] DOCUMENT TYPES: (MEETING ABSTRACTS OR EDITORIAL MATERIALS OR PROCEEDINGS PAPERS OR REVIEW ARTICLES)

AND LANGUAGE: (English)

2022 or 2021 or 2020 or 2019 or 2018 or 2017 or 2016 or 2015 or 2014 or 2013 or 2012 or 2011 or 2010 or 2009 or 2008 or 2007 or 2006 or 2005 or 2004 or 2003 or 2002 or 2001

(Note that the earliest record in this database was published in 2001).

## CENTRAL (via the Cochrane library) – CT.GOV and ICTRP registries

**Population**

#1 MeSH descriptor: [Asthma] explode all trees 12230

#2 asthma*:ti,ab 34031

#3 #1 OR #2 34688

**Intervention**

#4 (omalizumab):ti,ab,kw 1001

#5 (xolair):ti,ab,kw 187

#6 (mepolizumab):ti,ab,kw 373

#7 (nucala):ti,ab,kw 18

#8 (bosatria):ti,ab,kw 0

#9 (reslizumab):ti,ab,kw 131

#10 (cinqair):ti,ab,kw 2

#11 (cinqaero):ti,ab,kw 3

#12 (cinqaer):ti,ab,kw 0

#13 (cinquil):ti,ab,kw 1

#14 (benralizumab):ti,ab,kw 248

#15 (fasenra):ti,ab,kw 8

#16 (dupilumab):ti,ab,kw 724

#17 (dupixent):ti,ab,kw 22

#18 (tralokinumab):ti,ab,kw 124

#19 (lebrikizumab):ti,ab,kw 96

#20 (TNX-650):ti,ab,kw 8

#21 (tezepelumab):ti,ab,kw 104

#22 (brodalumab):ti,ab,kw 172

#23 (siliq):ti,ab,kw 1

#24 (kyntheum):ti,ab,kw 7

#25 (ligelizumab):ti,ab,kw 59

#26 (QGE031):ti,ab,kw 42

#27 (pitrakinra):ti,ab,kw 6

#28 (aerovant):ti,ab,kw 2

#29 #4 OR #5 OR #6 OR #7 OR #8 OR #9 OR #10 OR #11 OR #12 OR #13 OR #14 OR #15 OR #16 OR #17 OR #18 OR #19 OR #20 OR #21 OR #22 OR #23 OR #24 OR #25 OR #26 OR #27 OR #28

**Combination**

#30 #3 AND #29

**Language limit**

It was not possible to limit the search to English language records in this database.

**Publication year limit**

A filter was used to restrict the publication year from 1990 to Jan 2024.

# Narrative summary of evidence for predictors of response to biologics

**Table S2.** Inflammatory biomarkers as predictors of response to biological therapies for severe asthma.

| **Biologic** | **Exacerbations** | **FEV_1_** | **Asthma control** | **(m)OCS use/dosage** | **Clinical response outcome** |
| --- | --- | --- | --- | --- | --- |
| **Raised blood eosinophil counts** | | | | | |
| **Mepolizumab** | ***-*** | *-* | A large RCT and large observational study found that higher BEC levels are associated with significantly greater improvements in ACQ-5 scores^6,7^. Although the observational study did not provide quantitative data^7^, the RCT reported BEC ≥300 and ≥500 cells/µL are associated with a higher likelihood of achieving clinically meaningful improvements in ACQ-5 scores, with OR of 2.97 for both groups (95% CI: 1.85–4.74, and 1.60–5.53 respectively), compared to BEC ≥150 cells/µL (OR, 95%CI: 2.58 (1.74-3.82))^6^. Conversely, higher BEC levels are associated with decreased SGRQ response (reduction of ≥4 points). The likelihood of achieving response is equivalent for BEC ≥150 and ≥300 cells/µL, both with OR of 2.43. However, for BEC ≥500 cells/µL, the likelihood of response is significantly lower, with an OR of 2.21 (95% CI: 1.16–4.22)^6^. | - | Two small real-world studies found that higher BEC is significantly associated with clinical response^8,9^. The first study reported that every unit increase in BEC significantly reduces the likelihood of non-response by 14% (OR 0.86, 95% CI: 0.73–1.00)^8^. The second study reported that higher BEC levels have moderate predictive ability for distinguishing early responders from partial responders (AUC=0.79, 95% CI: 0.57–1), with an optimal cut-off of ≥580 cells/mm³ for identifying partial responders (sensitivity: 80%; specificity: 70%)^9^.  In contrast, another real-world small study indicated that BEC of ≥500/mm^3^ is not significantly associated with clinical response^10^. Additionally, one small real-world study showed that higher BEC is significantly associated with an increased likelihood of negative response (OR, 95%CI: 0.23 (0.06-0.98))^11^. |
| **Benralizumab** | - | - | - | **-** | A small real-world study found that higher BEC levels are strong predictors for identifying responders, achieving an AUC of 0.81 (95% CI: 0.56 to 1.00) with an optimal cut-off of 100/µL^12^. Additionally, a large real-world study showed that higher peak BEC in the year prior to benralizuamb is significantly associated with a higher likelihood of achieving super-response (OR, 95%CI: 3.89 (1.12-13.46)^13^. A model including peak BEC and other variables demonstrated moderate ability to distinguish super-responders from responders (AUC 76% (95% CI, 67%-85%))^13^.  In contrast, two large studies—one real-world cohort^13^ and one *post-hoc* analysis of a real-world cohort^14^—reported no significant association between higher BEC levels and clinical response. |
| **Dupilumab** | A large RCT found that higher BEC levels (≥300 cells/µL) are associated with a statistically significant 71.1% relative risk reduction in exacerbations compared to placebo, while patients with BEC <300 cells/µL experience a 45.5% reduction. Lower BEC levels (<150 cells/µL) indicate a significant 60.4% reduction, whereas BEC levels ≥150 cells/µL show a 58.2% relative risk reduction in severe exacerbations^15^. | A large RCT found that higher BEC levels (≥ 300 cells/µL) were associated with a significant and clinically meaningful improvement in pre-BD FEV_1_ (0.32L) compared to lower BEC levels (<300 cells/µL, 0.13L). Lower BEC groups (<150 cells/µL) showed marginally greater improvement (0.24L) than those with BEC >150 cells/µL (0.22L)^15^.  A post-hoc analysis of the same RCT showed that higher BEC levels (≥150 and ≥300 cells/µL) were associated with significant post-BD FEV_1_ improvements of 0.21L and 0.30L, respectively. Lower BEC groups (<150 and <300 cells/µL) did not significantly predict post-BD FEV_1_ improvement^16^. | **-** | A large RCT found that higher BEC levels are significant predictors of glucocorticoid dose reduction and achieving steroid-free status.  Patients with BEC >300 cells/µL experienced a 36.38% reduction in OCS dose, compared to a 21.33% reduction for those with BEC <300 cells/µL. Those with BEC >150 cells/µL had a 29.39% reduction, while patients below this threshold showed a 26.89% reduction that was not statistically significant.  Patients with BEC >300 cells/µL had a significantly higher likelihood of achieving a >50% reduction in OCS dose (OR 6.59 (2.12 to 20.42), compared to those with BEC < 300 cells/µL (OR 2.91 (1.28 to 6.63). Similarly, patients with BEC >150 cells/µL had significantly greater odds of achieving this reduction (OR 4.49 (2.04 to 9.85) than those below this threshold (OR 3.33 (0.97 to 11.48), although the latter was not statistically significant.  Patients with BEC >300 cells/µL were significantly more likely to reduce their glucocorticoid dose to <5 mg/d (OR 8.04 (2.71 to 23.82)), compared to those with BEC < 300 cells/µL (OR 3.12 (1.41 to 6.93)). Patients with BEC >150 cells/µL had a significantly lower likelihood of achieving this dose reduction (OR 4.29 (2.04 to 9.04)) than those below this threshold (OR 6.03 (1.70 to 21.44)), though the wide CI indicate an imprecise effect estimate.  Patients with BEC >300 cells/µL had a significantly higher likelihood of achieving steroid-free status (OR 4.07 (1.46 to 11.33)), compared to those with BEC < 300 cells/µL (OR 2.15 (0.96 to 4.81)). Similarly, patients with BEC >150 cells/µL were less likely to achieving steroid-free status (OR 2.73 (1.31 to 5.70)) than those below this threshold (OR 3.15 (0.93 to 10.73)), although the latter was not statistically significant^15^. | **-** |
| **Tezepelumab** | A large RCT found that high BEC (≥150 and ≥300 cells/µL) is associated with a statistically significant and clinically meaningful reduction in exacerbation rates, with decreases of 57% and 71%, respectively. Conversely, lower BEC (<150 and <300 cells/µL) is associated with a 35% and 12% increase in exacerbation rates, though these changes were not statistically significant^17^. | A large RCT found that high BEC levels (≥150 and ≥300 cells/µL) are associated with significantly greater and clinically meaningful improvements in pre-BD FEV_1_, with increases of 0.32 L and 0.37 L respectively. In contrast, lower BEC levels (<150 and <300 cells/µL) were associated with smaller FEV1 improvements of 0.16 L and 0.18 L, although these differences were not statistically significant^17^. | A large RCT showed that higher BEC (≥150 and ≥300 cells/µL) is not associated with significantly greater improvements in ACQ-6 scores compared to lower BEC^17^. | A large RCT found that high BEC (≥150 and ≥300 cells/µL) are associated with a significantly higher likelihood of achieving clinically meaningful reductions in mOCS, with OR of 2.58 (95% CI: 1.16–5.75) and 3.49 (95% CI: 1.16–10.49), respectively. In contrast, lower BEC (<150 and <300 cells/µL) is associated with lower likelihood of achieving OCS reduction, with OR of 0.4 (95% CI: 0.14–1.13) and 0.7 (95% CI: 0.33–1.47), although these findings were not statistically significant^17^. | **-** |
| **FeNO** | | | | | |
| **Mepolizumab** | - | **-** | - | **-** | A small observational study showed that each 10ppb increase in FeNO levels is not a significant predictor of clinical non-response^8^. |
| **Benralizumab** | - | **-** | - | **-** | A large real-world study showed that raised FeNO levels are not significantly associated with clinical response^13^. In contrast, a small real-world study found that elevated FeNO levels have moderate predictive ability for identifying responders, with an AUC of 0.76 (95% CI: 0.52-1.00) and an optimal cut-off of 40 ppb^12^.  A small real-world study demonstrated that elevated FeNO levels possess strong predictive ability for identifying super-responders, achieving an AUC of 0.856 with an optimal cut-off of 44 ppb (100% sensitivity and 72.7% specificity)^18^. |
| **Dupilumab** | A large RCT found that FeNO levels >25 to <50 ppb are associated with the greatest reduction in exacerbation risk, with a statistically significant and clinically meaningful decrease of 80.5%. In contrast, both lower (<25 ppb) and higher (>50 ppb) FeNO levels showed non-significant risk reductions of 29.6% and 46.8%, respectively^15^. | A large RCT and *post-hoc* analysis demonstrated that low FeNO levels (<25 ppb) are associated with significant improvements in pre- and post-BD FEV_1_ (ranging from 0.12-0.23 L)^15,16^. High FeNO levels (≥25 ppb) show inconsistent results; the *post-hoc* analysis indicated significant improvements in pre-BD FEV_1_ (0.20 L) but not post-BD FEV_1_ (0.24 L) ^16^, whereas the RCT reported statistically in-significant increases in pre-BD FEV_1_ for FeNO levels >25 to <50 ppb (0.23 L) and >50 ppb (0.28 L)^15^. | **-** | A large RCT found that FeNO levels >25 ppb are significantly associated with oral glucocorticoid dose reduction. Specifically, a 38.31% reduction was observed in the 25–50 ppb group and a 33.64% reduction in the >50 ppb group. In contrast, FeNO levels <25 ppb did not significantly predict glucocorticoid dose reduction^15^. | **-** |
| **Blood eosinophil counts + FeNO** | | | | | |
| **Dupilumab** | - | A *post-hoc* analysis of a large RCT found that high eosinophil counts (≥ 150 cells/µL) combined with low FeNO levels (< 25 ppb) are associated with statistically significant and clinically meaningful improvements of 0.24 L in both pre- and post-BD FEV_1_. In contrast, combinations involving lower eosinophil counts or higher FeNO levels yielded inconsistent and generally non-significant results, indicating limited predictive value for lung function improvement.  When high eosinophil counts (≥150 cells/µL) were combined with high FeNO levels (≥25 ppb), moderate improvements in FEV_1_ were observed, with LSMD of 0.20 L for pre-BD FEV_1_ (not statistically significant) and 0.24 L for post-BD FEV_1_ (statistically significant and clinically meaningful). Conversely, combinations of low eosinophils (<150 cells/µL) with either low FeNO (<25 ppb) or high FeNO (≥25 ppb) did not demonstrate statistically significant improvements in either pre- or post-BD FEV_1_^16^. | - | **-** | **-** |
| **Raised periostin levels** | | | | | |
| **Mepolizumab** | - | **-** | - | **-** | A small observational study showed that serum periostin levels have moderate predictive ability for distinguishing responders from non-responders (AUC 0.74). The best cut-off for predicting responders was 92.5 ng/mL (sensitivity, 83.3%; specificity, 73.3%)^19^. |
| **Raised peripheral neutrophils** | | | | | |
| **Mepolizumab** | - | **-** | - | **-** | A small observational study showed that peripheral neutrophil counts and neutrophil frequencies have moderate  predictive ability for distinguishing responders from non-responders (AUC 0.77 and 0.76, respectively). The best cutoffs for predicting responders were peripheral neutrophil counts of 4035 cells/mL (sensitivity, 75.0%; specificity, 73.3%), and neutrophil frequencies of 61.6% of white blood cells (sensitivity, 75.0%; specificity, 66.7%)^19^. |
| **T2 phenotype** | | | | | |
| **Mepolizumab** | - | A large real-world study showed that T2 phenotype (IgE >150 and/or Eos >300 and/or FeNO >25) and T2 phenotype_150 (IgE >150 and/or Eos >150 and/or FeNO >25) are not significant predictors of a ≥100ml FEV_1_ improvement^20^. | A large real-world study showed that T2 phenotype (IgE >150 and/or Eos >300 and/or FeNO >25) and T2 phenotype_150 (IgE >150 and/or Eos >150 and/or FeNO >25) are not significant predictors of ACT score improvement^20^. | **-** | **-** |
| **Th17 cells** | | | | | |
| **Benralizumab** | - | **-** | - | **-** | A small real-world study demonstrated that high Th17 cell frequencies have moderate predictive ability for distinguishing super-responders from non-responders, achieving an AUC of 0.768. The optimal cut-off for predicting super-responders was 4.77% Th17 cells, with 100% sensitivity and 54.6% specificity. The AUC for identifying responders was 0.733, with a cut-off of 4.57% Th17 cells (100% sensitivity and 56.3% specificity)^18^. |
| **ILC3** | | | | | |
| **Benralizumab** | - | **-** | - | **-** | A small real-world study showed that higher levels of ILC3 (Group 3 innate lymphoid cells) have moderate predictive ability for distinguishing responders from non-responders (AUC=0.713). The best cut-off for predicting responders was 11.45% of ILCs (sensitivity: 73.3%; specificity: 62.5%)^18^. |
| **Atopic status** | | | | | |
| **Mepolizumab** | - | **-** | - | A large observational study showed that atopic status is not significantly associated with improvement in ACQ-5 scores^7^. | A small real-world study reported that atopy is not significantly associated with clinical super-response^21^. |
| **Benralizumab** | - | **-** | - | **-** | In a *post-hoc* analysis of a large real-world cohort, and a large real-world study, non-atopic and atopic phenotype were not significantly associated with clinical response^14^ and super-response^13^ respectively. |

Key: Green = investigated variable predicts positive biologic response, red = investigated variable predicts negative biologic response, blue = no association between investigated variable and biologic response.

Abbreviations: BEC: Blood Eosinophil Counts; FEV_1_: Forced expiratory volume in one second; mOCS: maintenance oral corticosteroids; ACQ: Asthma Control Questionnaire; FeNO: Fractional exhaled nitric oxide; CI: Confidence Intervals; OR: Odds ratios; AUC, Area Under the Curve.

**Table S3.** Lung function parameters as predictors of response to biological therapies for severe asthma.

| **Biologic** | **Exacerbations** | **Clinical response outcome** |
| --- | --- | --- |
| **Normal FEV_1_** | | |
| **Mepolizumab** | **-** | A small real-world study showed that FEV_1_% is not significantly associated with super-response^21^, whilst another reported that FEV_1_ L and FEV_1_% predicted are not significantly associated with non-response^8^. |
| **Benralizumab** | **-** | Two large studies (real-world cohort and *post-hoc* analysis respectively) and one small real-world study, found that FEV_1_ variables (such as FEV_1_<60%, FEV_1_% predicted, and pre-BD FEV_1_% predicted) are not significantly associated with clinical response^13,14,22^. The large real-world study showed that FEV_1_% predicted is weakly associated with a higher likelihood of achieving super-response, but the effect size is small (OR of 1.02 with a 95% CI of 1.00–1.05)^13^. |
| **Normal FEV_1_/FVC<LLN** | | |
| **Mepolizumab** | A large real-world study showed that FEV_1_/FVC<LLN is not significantly associated with a reduction in exacerbations^20^. | **-** |
| **Benralizumab** | A small real-world study showed that FEV_1_/FVC<LLN is not significantly associated with a reduction in exacerbations^20^. | **-** |

Key: Green = investigated variable predicts positive biologic response, red = investigated variable predicts negative biologic response, blue = no association between investigated variable and biologic response.

Abbreviations: FEV_1_: Forced expiratory volume in one second; FVC: Forced vital capacity; mOCS: maintenance oral corticosteroids; ACQ: Asthma Control Questionnaire; CI: Confidence Intervals; OR: Odds ratios.

**Table S4.** Clinical parameters as predictors of response to biological therapies for severe asthma.

| **Biologic** | **Exacerbations** | **FEV_1_** | **Asthma control** | **(m)OCS use/dosage** | **Clinical response outcome** |
| --- | --- | --- | --- | --- | --- |
| **Worse exacerbation history** | | | | | |
| **Mepolizumab** | - | **-** | - | **-** | A small real-world study showed that exacerbation history is not predictive of non-response^8^. In contrast, another small real-world study reported that a higher exacerbation rate at baseline is predictive of super-response (OR = 1.49, 95% CI: 1.05–2.12). The model showed good predictive accuracy, with an AUC of 0.811, indicating that it correctly distinguished super-responders from non-super-responders about 81.1% of the time^21^. |
| **Benralizumab** | - | **-** | - | **-** | A large real-world study and a *post-hoc* analysis of a real-world cohort found no significant association between exacerbation history and response^13,14^ or super-response^13^. |
| **OCS use/dose** | | | | | |
| **Mepolizumab** | A large real-world study found that patients not on mOCS and those on lower doses (<10 mg/d) had a significantly reduced risk of exacerbations, including those requiring hospitalisation or emergency visits, compared to patients on mOCS and higher doses (≥10 mg/d).  Patients not on mOCS experienced fewer clinically significant exacerbations (RR (95%CI): 0.28 (0.25, 0.31)) than those on mOCS (RR (95%CI): 0.30 (0.26, 0.35)). Among mOCS users, lower dose was associated with a lower exacerbation rate (RR (95%CI): 0.25 (0.20, 0.32)) compared to higher doses (RR (95%CI): 0.36 (0.29, 0.44)). The likelihood of experiencing no exacerbations was highest in patients not on mOCS (OR (95%CI): 16.3 (11.2, 23.7)) and those on lower doses (OR (95%CI): 19.2 (9.4, 39.2)), compared to those on higher doses (OR (95%CI): 6.2 (3.6, 10.4)).  Fewer exacerbations required hospital or ER visits among patients not on mOCS or on lower mOCS doses. Lower-dose users had a rate ratio of 0.21 (95%CI: 0.14, 0.32), compared to 0.25 (95%CI: 0.17, 0.35) for higher-dose users.  Patients on lower mOCS doses had a higher likelihood of avoiding exacerbation-related hospitalisations (RR (95%CI): 3.78 (1.82, 7.83)) compared to those on higher doses (RR (95%CI): 3.48 (2.03, 5.94))^23^. | **-** | - | A large real-world study reported that mOCS use at baseline significantly decreases the likelihood of achieving OCS-free status after 6 months of treatment by 90.5% (OR, 95% CI: 0.095 (0.040 - 0.227)^24^. | A small real-world study showed that higher OCS daily dose is associated with a higher likelihood of non-response (OR, 95%CI: 1.15 (1.07–1.24)^8^. Conversely, two other small real-world studies reported that mOCS use is not significantly associated with clinical response^11,21^. |
| **Benralizumab** | - | **-** | - | **-** | A large real-world study and a *post-hoc* analysis of a real-world cohort found that OCS use and dosage are not significant predictors of clinical response^13,14^. OCS use significantly reduced the likelihood of achieving super-response by 68%^13^. However, when combined with a panel of other variables associated with super-response, the model demonstrated moderate predictive ability (AUC: 76%, 95% CI: 67-85)^13^. Higher OCS dose at 3 months was significantly associated with negative response; for each additional mg/day of OCS, the odds of a positive response decreased by 11%^14^. |
| **Dupilumab** | A *post-hoc* analysis of a large RCT found that patients on low dose OCS (<10 mg/d) have a statistically significant and clinically meaningful greater reduction in severe exacerbations (RR 0.29, 95% CI 0.13-0.64) compared to those on high OCS dose (≥10 mg/d) (RR 0.52, 95% CI 0.31-0.86^25^. | A *post-hoc* analysis of a large RCT showed that patients on a low baseline dose of OCS (<10 mg/d) have a significantly greater and clinically meaningful improvement in post-BD FEV_1_ (0.20 L, 95% CI 0.05-0.35) compared to those on high OCS dose (≥10 mg/d) who had an improvement of 0.18 L (95% CI 0.02-0.34).  In contrast, for pre-BD FEV_1_, patients on a high baseline OCS dose (≥10 mg/d) had a significantly greater and clinically meaningful improvement (0.26 L (95% CI: 0.09-0.43) compared to those on low dose OCS (<10 mg/d) , who showed a more modest and statistically non-significant improvement of 0.15 L (95% CI: -0.04 to 0.33)^25^. | **-** | A *post-hoc* analysis of a large RCT demonstrated that patients on low OCS dose (<10 mg/d) are significantly more likely to achieve steroid-free status after 24 weeks (OR, 95% CI: 3.75 (1.40–10.01) compared to those on high OCS dose (≥10 mg/d) (OR, 95% CI: 2.32 (1.00–5.38))^25^. | - |
| **Increased ICS dose** | | | | | |
| **Benralizumab** | - | **-** | - | **-** | A *post-hoc* analysis of a large real-world study showed that ICS dose at baseline is not predictive of clinical response^14^. |
| **Biologic naïve** | | | | | |
| **Benralizumab** | - | **-** | - | **-** | A *post-hoc* analysis of a large real-world study showed that biologic naïve patients are significantly more likely to achieve clinical response (OR 95% CI: 3.87 (1.83-8.17))^14^. |
| **Increased number of respiratory medicines** | | | | | |
| **Mepolizumab** | - | **-** | - | A large real-world study reported that number of respiratory medications at baseline is not a significant predictor of becoming OCS-free after 6 months of treatment^24^. | **-** |
| **Better asthma control** | | | | | |
| **Mepolizumab** | - | **-** | - | A large real-world study showed that the likelihood of achieving OCS free status after 6 months of treatment significantly increased by 11.1% for each unit increase in ACT score i.e. better asthma control^24^. | A small real-world study reported that a better ACT score is not significantly associated with clinical non-response^8^. Likewise, two other small real-world studies found that better asthma control (lower ACQ-6 score) at baseline is associated with a significantly higher likelihood of achieving clinical response (OR=4.65 (95% CI: 1.51–14.29) and OR=0.73 (95% CI: 0.60–0.89)), and super-response (OR=3.40 (95% CI: 1.17–9.90) and OR=0.76 (95% CI: 0.58–0.98))^21,26^.  A model including ACQ-6 score demonstrated moderate^26^ to good^21^ predictive ability for distinguishing responders from non-responders (AUC values ranging from 0.735 to 0.859). Additionally, a model that included asthma control along with NP demonstrated moderate predictive ability for distinguishing super-responders from non-super-responders (AUC=0.78)^26^. |
| **Benralizumab** | - | **-** | - | **-** | A large real-world study and a *post-hoc* analysis of a large real-world cohort showed that better asthma control (lower ACQ-6 score) is not a significant predictor of response^13,14^ or super-response^13^. However, it was unclear which version of the ACQ-6 was used in each study. |
|  |  |  |  |  | A *post-hoc* analysis of a large real-world study reported that better asthma control (lower ACQ-6 score) 3 months post treatment significantly increases the likelihood of achieving clinical response by 68% (OR, 95% CI: 0.32 (0.19–0.54))^14^. |
| **Better quality of life** | | | | | |
| **Mepolizumab** | - | **-** | - | **-** | A small real-world study reported that QoL is not significantly associated with clinical response^21^. |
| **Adult-onset asthma** | | | | | |
| **Mepolizumab** | - | **-** | A large observational study found that later age of asthma onset (≥40 years) is associated with greater improvement in ACQ-5 scores compared to earlier onset (<40 years), although the improvement did not reach the MCID^7^. | A large real-world study reported that later age of asthma onset is associated with a marginally increased likelihood of achieving OCS-free status after 6 months, but it is not clinically significant (OR (95% CI): 1.03 (1.00 - 1.05))^24^. | A small real-world study reported that adult-onset asthma is not significantly associated with clinical response^21^. |
| **Benralizumab** | - | **-** | - | **-** | Two large studies (real-world cohort and *post-hoc* analysis of real-world study) found that adult-onset disease is not a significant predictor of clinical response^13,14^ or super-response^13^. |
| **Childhood-onset asthma** | | | | | |
| **Benralizumab** | - | **-** | - | ***-*** | A small real-world study showed that childhood-onset asthma increases the likelihood of achieving clinical response (OR (95% CI): 7.17 (1.10-46.9), but the wide CI indicate uncertainty about effect estimate^22^. |

Key: Green = investigated variable predicts positive biologic response, red = investigated variable predicts negative biologic response, blue = no association between investigated variable and biologic response.

Abbreviations: FEV_1_: Forced expiratory volume in one second; QoL: Quality of Life; mOCS: maintenance oral corticosteroids; ACQ: Asthma Control Questionnaire; NP: Nasal polyposis; CI: Confidence Intervals; OR: Odds ratios; AUC: Area Under the Curve.

**Table S5.** Co-morbidities as predictors of response to biological therapies for severe asthma.

| **Biologic** | **Exacerbations** | **Asthma control** | **Clinical response outcome** |
| --- | --- | --- | --- |
| **Allergic disease** | | | |
| **Mepolizumab** | - | - | A small real-world study showed that allergy is not significantly associated with clinical response^10^. |
| **Chronic rhinosinusitis** | | | |
| **Benralizumab** | - | - | A *post-hoc* analysis of a large real-world study found that co-morbid chronic rhinosinusitis is not a significant predictor of clinical response^14^. |
| **NARES** | | | |
| **Mepolizumab** | - | - | A small real-world study showed that NARES is not significantly associated with clinical response^10^. |
| **Nasal Polyps** | | | |
| **Mepolizumab** | **-** | A large real-world study found that NP is not significantly associated with improvement in ACT score^20^. | A small real-world study reported that NP is not a significant predictor of clinical non-response^8^. In contrast, a small real-world study demonstrated that a model including NP and other variables has moderate predictive ability for identifying responders (AUC = 0.753). Additionally, NP is significantly associated with higher likelihood of achieving super-response, although the CI are wide (OR = 4.19, 95% CI: 1.21–14.49). The optimal model for super-response, which included NP and baseline ACQ, had moderate discriminative ability (AUC=0.78)^26^. |
| **Benralizumab** | - | A small real-world study showed that having NP significantly decreases the likelihood of achieving ACT score improvement (OR 95% CI: 0.14 (0.02 - 0.99))^20^, although it is unclear whether this is clinically meaningful. | Two large studies showed that NP is not a significant predictor of clinical (super) response^13,14^. |
| **Bronchiectasis** | | | |
| **Mepolizumab** | - | - | Two small real-world studies showed that having bronchiectasis is not a significant predictor of clinical response^10,21^. |
| **GERD** | | | |
| **Mepolizumab** | A large real-world study showed that GERD is not significantly associated with reduction in exacerbations^20^. | A large real-world study showed that GERD is not significantly associated with ACT score improvement^20^. | A small real-world study showed that GERD is not significantly associated with clinical response^10^. |
| **Benralizumab** | A small real-world study found that GERD is not significantly associated with reduction in exacerbations^20^. | A small real-world study found that GERD is not significantly associated with ACT score improvement^20^. | - |
| **Obesity** | | | |
| **Mepolizumab** | - | - | A small real-world study showed that obesity is not significantly associated with clinical response^10^. |
| **Osteoporosis** | | | |
| **Mepolizumab** | - | A large real-world study showed that osteoporosis is not significantly associated with ACT score improvement^20^. | - |
| **Benralizumab** | - | A small real-world study showed that osteoporosis significantly decreases the likelihood of achieving ACT score improvement (OR 95% CI: 0.08 (0.007 - 0.93)))^20^, although it is unclear whether this is clinically meaningful. | - |
| **Dysfunctional breathing** | | | |
| **Mepolizumab** | - | - | A small real-world study reported that dysfunctional breathing is not significantly associated with clinical response^21^. |
| **Mental health conditions** | | | |
| **Mepolizumab** | - | - | A small real-world study reported that Depression, Anxiety, and Total HADS score are not significantly associated with clinical response^21^. |

Key: Green = investigated variable predicts positive biologic response, red = investigated variable predicts negative biologic response, blue = no association between investigated variable and biologic response.

Abbreviations: BMI: Body Mass Index; FEV_1_: Forced expiratory volume in one second; QoL: Quality of Life; mOCS: maintenance oral corticosteroids; ACQ: Asthma Control Questionnaire; NP: Nasal polyposis; GERD: Gastroesophageal reflux disease; HADS: Hospital Anxiety and Depression Scale; CI: Confidence Intervals; OR: Odds ratios.

**Table S6.** Socio-demographic characteristics as predictors of response to biological therapies for severe asthma.

| **Biologic** | **Exacerbations** | **FEV_1_** | **Asthma control** | **(m)OCS use/dosage** | **Clinical response outcome** |
| --- | --- | --- | --- | --- | --- |
| **Age** | | | | | |
| **Mepolizumab** | - | A large real-world study found older age (>50 years) is not significantly associated with FEV_1_ improvement of ≥100 mL^20^. | A large observational study found that age is not a significant predictor of improvement in ACQ-5 score^7^, but it was unclear whether this was older or younger age. | - | A small observational study reported that younger age is a borderline predictor of clinical non-response (OR 0.93, 95% CI: 0.86–1.00 per year)^8^.  A small real-world study found that for every 5-year increase in age, the likelihood of achieving clinical response significantly increases by 24% (OR=1.24 (95% CI=1.03-1.50)^26^. In contrast, three small real-world cohort studies found no significant association between older age and response^10,11^ or super-response^26^. |
| **Benralizumab** | - | A small real-world study found no significant association between older age (>50 years) and FEV_1_ improvement of ≥100 mL^20^. | - | - | Two large studies^13,14^ (a real-world cohort and *post-hoc* analysis of a real-world study respectively), and one small observational study^22^ found that age is not a significant predictor of clinical response. However, age thresholds were not defined in any of the included studies. |
| **Gender** | | | | | |
| **Mepolizumab** | - | - | A large observational study showed that male gender is predictive of lower ACQ-5 score improvement^7^. | - | Two small real-world studies showed that female gender is not significantly associated with negative (OR, 95% CI: 1.70 (0.62–4.67))^8^ or positive clinical response (OR, 95% CI: 1.11 (0.06 – 2.85))^10^. |
| **Benralizumab** | - | - | - | - | A small real-world study found that female gender is not significantly associated with negative response^22^, whilst a large real-world study found that being female is not associated with positive clinical response^13^. A *post-hoc* analysis of a large real-world study showed that male gender is not a significant predictor of response^14^. Given the heterogeneity in the variable and response outcome definitions across included studies, it is unclear whether gender is predictive of response. |
| **High BMI** | | | | | |
| **Mepolizumab** | A large real-world study demonstrated that patients with BMI >30 kg/m² are significantly more likely to experience more exacerbations (defined as ≥1 in 12 months) i.e. negative response (OR, 95% CI: 2.30 (1.00 - 5.29)^20^. | - | A large observational study reported that BMI ≥ 30 kg/m² is significantly associated with reduced ACQ-5 response compared to BMI < 30 kg/m²^7^. | A large real-world study found that the likelihood of achieving steroid-free status after 6 months of treatment decreased by 7.5% for each BMI unit increase (OR: 0.93, 95% CI: 0.87 - 0.98)^24^. Although statistically significant, this reflects a modest effect size. | Four small real-world studies demonstrated that higher BMI is not significantly associated with clinical response^8,10,11,21^. However, three of these studies did not report comparison between different BMI thresholds. |
| **Benralizumab** | - | - | - | - | A small real-world study showed that higher BMI is not associated with negative response^22^. Two large studies (real-world cohort and *post-hoc* analysis of observational cohort respectively) found that higher BMI is not a predictor of clinical response or super-response^13,14^. However, the data were not statistically significant. |
| **Smoking status** | | | | | |
| **Mepolizumab** | - | - | A large observational study considered smoking status (ex-/current or never)in a multiple linear regression analysis, but it was not associated with a statistically significant improvement in ACQ-5 score, and thus not included in the multivariate model^7^. | - | Two small real-world studies found that smoking is not significantly associated with clinical response^10,21^. However, only one study reported quantitative data (OR 2.66, 95% CI 0.16 – 6.84)^10^. |
| **Benralizumab** | - | - | - |  | A *post-hoc* analysis of a large real-world study found no significant association between being an ex-smoker and clinical response (OR = 0.71, 95% CI: 0.37–1.38)^14^. |
| **Geographic region** | | | | | |
| **Mepolizumab** | - | - | A large real-world study found no significant association between living in rural vs urban areas and improvement in ACT score (OR 2.00, 95% CI 0.55 - 7.34)^20^. | - | - |
| **Benralizumab** | - | - | A small real-world study found no significant association between living in rural vs urban areas and improvement in ACT score (OR 1.00, 95% CI 0.44 - 4.41) ^20^. | - | - |

Key: Green = investigated variable predicts positive biologic response, red = investigated variable predicts negative biologic response, blue = no association between investigated variable and biologic response.

Abbreviations: BMI: Body Mass Index; FEV_1_: Forced expiratory volume in one second; mOCS: maintenance oral corticosteroids; ACQ: Asthma Control Questionnaire; NP: Nasal polyposis; GERD: Gastroesophageal reflux disease; HADS: Hospital Anxiety and Depression Scale; CI: Confidence Intervals; OR: Odds ratios.

# PRISMA checklist

**Table S7.** Preferred Reporting Items for Systematic Reviews and Meta-Analyses (PRISMA) checklist.

| **Section and Topic** | **Item #** | **Checklist item** | **Location where item is reported** |
| --- | --- | --- | --- |
| **TITLE** | | |  |
| Title | 1 | Identify the report as a systematic review. | Title |
| **ABSTRACT** | | |  |
| Abstract | 2 | See the PRISMA 2020 for Abstracts checklist. | NA |
| **INTRODUCTION** | | |  |
| Rationale | 3 | Describe the rationale for the review in the context of existing knowledge. | Background |
| Objectives | 4 | Provide an explicit statement of the objective(s) or question(s) the review addresses. | Background |
| **METHODS** | | |  |
| Eligibility criteria | 5 | Specify the inclusion and exclusion criteria for the review and how studies were grouped for the syntheses. | Methods: eligibility criteria (section 2.2) |
| Information sources | 6 | Specify all databases, registers, websites, organisations, reference lists and other sources searched or consulted to identify studies. Specify the date when each source was last searched or consulted. | Methods: search strategy (section 2.1) |
| Search strategy | 7 | Present the full search strategies for all databases, registers and websites, including any filters and limits used. | Online supplement (section II) |
| Selection process | 8 | Specify the methods used to decide whether a study met the inclusion criteria of the review, including how many reviewers screened each record and each report retrieved, whether they worked independently, and if applicable, details of automation tools used in the process. | Methods: study selection (section 2.3) |
| Data collection process | 9 | Specify the methods used to collect data from reports, including how many reviewers collected data from each report, whether they worked independently, any processes for obtaining or confirming data from study investigators, and if applicable, details of automation tools used in the process. | Methods: data extraction (section 2.3); quality appraisal strategy (section 2.4); analysis and synthesis of results (section 2.5). |
| Data items | 10a | List and define all outcomes for which data were sought. Specify whether all results that were compatible with each outcome domain in each study were sought (e.g. for all measures, time points, analyses), and if not, the methods used to decide which results to collect. | Methods: eligibility criteria (section 2.2) |
|  | 10b | List and define all other variables for which data were sought (e.g. participant and intervention characteristics, funding sources). Describe any assumptions made about any missing or unclear information. | Methods: study selection (section 2.3) |
| Study risk of bias assessment | 11 | Specify the methods used to assess risk of bias in the included studies, including details of the tool(s) used, how many reviewers assessed each study and whether they worked independently, and if applicable, details of automation tools used in the process. | Methods: quality appraisal strategy (section 2.4). |
| Effect measures | 12 | Specify for each outcome the effect measure(s) (e.g. risk ratio, mean difference) used in the synthesis or presentation of results. | NA |
| Synthesis methods | 13a | Describe the processes used to decide which studies were eligible for each synthesis (e.g. tabulating the study intervention characteristics and comparing against the planned groups for each synthesis (item #5)). | NA |
|  | 13b | Describe any methods required to prepare the data for presentation or synthesis, such as handling of missing summary statistics, or data conversions. | NA |
|  | 13c | Describe any methods used to tabulate or visually display results of individual studies and syntheses. | NA |
|  | 13d | Describe any methods used to synthesize results and provide a rationale for the choice(s). If meta-analysis was performed, describe the model(s), method(s) to identify the presence and extent of statistical heterogeneity, and software package(s) used. | Methods: analysis and synthesis of results (section 2.5). |
|  | 13e | Describe any methods used to explore possible causes of heterogeneity among study results (e.g. subgroup analysis, meta-regression). | NA |
|  | 13f | Describe any sensitivity analyses conducted to assess robustness of the synthesized results. | NA |
| Reporting bias assessment | 14 | Describe any methods used to assess risk of bias due to missing results in a synthesis (arising from reporting biases). | NA |
| Certainty assessment | 15 | Describe any methods used to assess certainty (or confidence) in the body of evidence for an outcome. | Methods: quality appraisal strategy (section 2.4); Table S1. |
| **RESULTS** | | |  |
| Study selection | 16a | Describe the results of the search and selection process, from the number of records identified in the search to the number of studies included in the review, ideally using a flow diagram. | Figure 1 |
|  | 16b | Cite studies that might appear to meet the inclusion criteria, but which were excluded, and explain why they were excluded. | Figure 1 |
| Study characteristics | 17 | Cite each included study and present its characteristics. | Table 1 |
| Risk of bias in studies | 18 | Present assessments of risk of bias for each included study. | Table 2 |
| Results of individual studies | 19 | For all outcomes, present, for each study: (a) summary statistics for each group (where appropriate) and (b) an effect estimate and its precision (e.g. confidence/credible interval), ideally using structured tables or plots. | NA |
| Results of syntheses | 20a | For each synthesis, briefly summarise the characteristics and risk of bias among contributing studies. | Results: study characteristics (section 3.1); risk of bias in included studies (section 3.2) |
|  | 20b | Present results of all statistical syntheses conducted. If meta-analysis was done, present for each the summary estimate and its precision (e.g. confidence/credible interval) and measures of statistical heterogeneity. If comparing groups, describe the direction of the effect. | Figure 2 and 3. |
|  | 20c | Present results of all investigations of possible causes of heterogeneity among study results. | NA |
|  | 20d | Present results of all sensitivity analyses conducted to assess the robustness of the synthesized results. | NA |
| Reporting biases | 21 | Present assessments of risk of bias due to missing results (arising from reporting biases) for each synthesis assessed. | NA |
| Certainty of evidence | 22 | Present assessments of certainty (or confidence) in the body of evidence for each outcome assessed. | Results: sections 3.3 to 3.7; Tables 3 to 7. |
| **DISCUSSION** | | |  |
| Discussion | 23a | Provide a general interpretation of the results in the context of other evidence. | Discussion (section 4) |
|  | 23b | Discuss any limitations of the evidence included in the review. | Discussion: strengths and limitations (section 4.1) |
|  | 23c | Discuss any limitations of the review processes used. | Discussion: strengths and limitations (section 4.1) |
|  | 23d | Discuss implications of the results for practice, policy, and future research. | Discussion: Implications for research and practice (section 4.2) |
| **OTHER INFORMATION** | | |  |
| Registration and protocol | 24a | Provide registration information for the review, including register name and registration number, or state that the review was not registered. | Methods (section 2) |
|  | 24b | Indicate where the review protocol can be accessed, or state that a protocol was not prepared. | Methods (section 2) |
|  | 24c | Describe and explain any amendments to information provided at registration or in the protocol. | NA |
| Support | 25 | Describe sources of financial or non-financial support for the review, and the role of the funders or sponsors in the review. | Other information: funding |
| Competing interests | 26 | Declare any competing interests of review authors. | Other information: conflict of interests |
| Availability of data, code and other materials | 27 | Report which of the following are publicly available and where they can be found: template data collection forms; data extracted from included studies; data used for all analyses; analytic code; any other materials used in the review. | Online supplement |

*From:*  Page MJ, McKenzie JE, Bossuyt PM, Boutron I, Hoffmann TC, Mulrow CD, et al. The PRISMA 2020 statement: an updated guideline for reporting systematic reviews. BMJ 2021;372:n71. doi: 10.1136/bmj.n7

#

# Reference list

1. Schatz M, Kosinski M, Yarlas AS, Hanlon J, Watson ME, Jhingran P. The minimally important difference of the Asthma Control Test. *Journal of Allergy and Clinical Immunology*. Oct 2009;124(4):719-723.

2. Juniper EF, O'Byrne PM, Roberts JN. Measuring asthma control in group studies: do we need airway calibre and rescue beta2-agonist use? *Respir Med*. May 2001;95(5):319-23. doi:10.1053/rmed.2001.1034

3. Juniper EF, Gruffydd-Jones K, Ward S, Svensson K. Asthma Control Questionnaire in children: validation, measurement properties, interpretation. *European Respiratory Journal*. Dec 36(6):1410-1416.

4. Jones PW. St. George's Respiratory Questionnaire: MCID. *Copd*. Mar 2005;2(1):75-9. doi:10.1081/copd-200050513

5. Agache I, Rocha C, Beltran J, et al. Efficacy and safety of treatment with biologicals (benralizumab, dupilumab and omalizumab) for severe allergic asthma: A systematic review for the EAACI Guidelines - recommendations on the use of biologicals in severe asthma. *Allergy*. May 2020;75(5):1043-1057. doi:10.1111/all.14235

6. Chupp GL, Bradford ES, Albers FC, et al. Efficacy of mepolizumab add-on therapy on health-related quality of life and markers of asthma control in severe eosinophilic asthma (MUSCA): a randomised, double-blind, placebo-controlled, parallel-group, multicentre, phase 3b trial. *Lancet Respir Med*. May 2017;5(5):390-400. doi:10.1016/s2213-2600(17)30125-x

7. Harvey ES, Langton D, Katelaris C, et al. Mepolizumab effectiveness and identification of super-responders in severe asthma. *EUROPEAN RESPIRATORY JOURNAL*. MAY-1 55(5)doi:doi:

8. Caminati M, Marcon A, Guarnieri G, et al. Benralizumab Efficacy in Late Non-Responders to Mepolizumab and Variables Associated with Occurrence of Switching: A Real-Word Perspective. *J Clin Med*. Feb 24 2023;12(5)doi:10.3390/jcm12051836

9. Bergantini L, d'Aless, ro M, et al. Personalized Approach of Severe Eosinophilic Asthma Patients Treated with Mepolizumab and Benralizumab. *International archives of allergy and immunology*. 2020 2020;181(10):746-753. doi:doi:

10. Crimi C, Campisi R, Cacopardo G, et al. Real-life effectiveness of mepolizumab in patients with severe refractory eosinophilic asthma and multiple comorbidities. *The World Allergy Organization journal*. 2020 2020;13(9):100462. doi:doi:

11. Reilly C, Raja A, Anilkumar P, et al. The clinical effectiveness of mepolizumab treatment in severe eosinophilic asthma; outcomes from four years cohort evaluation. *J Asthma*. Jun 2024;61(6):561-573. doi:10.1080/02770903.2023.2294908

12. Watanabe H, Shirai T, Hirai K, et al. Blood eosinophil count and FeNO to predict benralizumab effectiveness in real-life severe asthma patients. *Journal of Asthma*. 2021 2021;doi:doi:

13. Kavanagh JE, Hearn AP, Dhariwal J, et al. Real-World Effectiveness of Benralizumab in Severe Eosinophilic Asthma. *CHEST*. FEB 159(2):496-506. doi:doi:

14. Kroes JA, de Jong K, Hashimoto S, et al. Clinical response to benralizumab can be predicted by combining clinical outcomes at 3 months with baseline characteristics. *ERJ Open Res*. Mar 2023;9(2)doi:10.1183/23120541.00559-2022

15. Rabe KF, Nair P, Brusselle G, et al. Efficacy and Safety of Dupilumab in Glucocorticoid-Dependent Severe Asthma. *N Engl J Med*. Jun 28 2018;378(26):2475-2485. doi:10.1056/NEJMoa1804093

16. Rabe KF, Nair P, Maspero JF, et al. The effect of dupilumab on lung function parameters in patients with oral corticosteroid-dependent severe asthma. *Respiratory Medicine: X*. 2020 2020;2:100010. doi:doi:

17. Wechsler ME, Menzies-Gow A, Brightling CE, et al. Evaluation of the oral corticosteroid-sparing effect of tezepelumab in adults with oral corticosteroid-dependent asthma (SOURCE): a randomised, placebo-controlled, phase 3 study. *The Lancet Respiratory medicine*. 2022 2022;doi:doi:

18. Sandhu Y, Harada N, Sasano H, et al. Pretreatment Frequency of Circulating Th17 Cells and FeNO Levels Predicted the Real-World Response after 1 Year of Benralizumab Treatment in Patients with Severe Asthma. *Biomolecules*. Mar 15 2023;13(3)doi:10.3390/biom13030538

19. Sasano H, Harada N, Harada S, et al. Pretreatment circulating MAIT cells, neutrophils, and periostin predicted the real-world response after 1-year mepolizumab treatment in asthmatics. *Allergol Int*. Jan 2024;73(1):94-106. doi:10.1016/j.alit.2023.06.001

20. Bilò MB, Martini M, Antonicelli L, et al. Severe asthma: follow-up after one year from the Italian Registry on Severe Asthma (IRSA). *Eur Ann Allergy Clin Immunol*. Sep 2023;55(5):199-211. doi:10.23822/EurAnnACI.1764-1489.304

21. Fong WCG, Azim A, Knight D, et al. Real-world Omalizumab and Mepolizumab treated difficult asthma phenotypes and their clinical outcomes. *CLINICAL AND EXPERIMENTAL ALLERGY*. AUG 51(8):1019-1032. doi:doi:

22. Al-Ahmad M, Ali A, Maher A. Factors influencing poor response to type 2 targeted therapies in severe asthma: a retrospective cohort study. *BMC Pulm Med*. Dec 5 2023;23(1):490. doi:10.1186/s12890-023-02786-w

23. Pilette C, Canonica GW, Chaudhuri R, et al. REALITI-A study: Real-world oral corticosteroid-sparing effect of mepolizumab in severe asthma. *The journal of allergy and clinical immunology In practice*. 2022 2022;doi:doi:

24. Thomas D, Harvey ES, McDonald VM, et al. Mepolizumab and Oral Corticosteroid Stewardship: Data from the Australian Mepolizumab Registry. *The journal of allergy and clinical immunology In practice*. 2021 2021;9(7):2715-2724.e5. doi:doi:

25. Domingo C, Maspero JF, Castro M, et al. Dupilumab Efficacy in Steroid-Dependent Severe Asthma by Baseline Oral Corticosteroid Dose. *The journal of allergy and clinical immunology In practice*. 2022 2022;10(7):1835-1843. doi:doi:

26. Kavanagh JE, d'Ancona G, Elstad M, et al. Real-World Effectiveness and the Characteristics of a "Super-Responder" to Mepolizumab in Severe Eosinophilic Asthma. *CHEST*. AUG 158(2):491-500. doi:doi:
